# Aberrant Gene Expression Profiling in Men With Sertoli Cell-Only Syndrome

**DOI:** 10.3389/fimmu.2022.821010

**Published:** 2022-06-27

**Authors:** Tong Chen, Yichun Wang, Linlin Tian, Xuejiang Guo, Jiadong Xia, Zengjun Wang, Ninghong Song

**Affiliations:** ^1^ Department of Urology, The First Affiliated Hospital of Nanjing Medical University, Nanjing, China; ^2^ Department of Microbiology Laboratory, Nanjing Municipal Center for Disease Control and Prevention, Nanjing, China; ^3^ State Key Laboratory of Reproductive Medicine, Nanjing Medical University, Nanjing, China; ^4^ The Affiliated Kezhou People’s Hospital of Nanjing Medical University, Kezhou, China

**Keywords:** cell cycle, functional enrichment, hub genes, immune cells, inflammation, Sertoli cell-only syndrome

## Abstract

Sertoli cell-only syndrome (SCOS) is the most severe and common pathological type of non-obstructive azoospermia. The etiology of SCOS remains largely unknown to date despite a handful of studies reported in this area. According to the gene expression of testicular tissue samples in six datasets from the Gene Expression Omnibus, we detected 1441 differentially expressed genes (DEGs) between SCOS and obstructive azoospermia (OA) testicular tissue samples. Enriched GO terms and KEGG pathways for the downregulated genes included various terms and pathways related to cell cycle and reproduction, while the enrichment for the upregulated genes yielded many inflammation-related terms and pathways. In accordance with the protein-protein interaction (PPI) network, all genes in the most critical module belonged to the downregulated DEGs, and we obtained nine hub genes, including CCNB1, AURKA, CCNA2, BIRC5, TYMS, UBE2C, CDC20, TOP2A, and OIP5. Among these hub genes, six were also found in the most significant SCOS-specific module obtained from consensus module analysis. In addition, most of SCOS-specific modules did not have a consensus counterpart. Based on the downregulated genes, transcription factors (TFs) and kinases within the upstream regulatory network were predicted. Then, we compared the difference in infiltrating levels of immune cells between OA and SCOS samples and found a significantly higher degree of infiltration for most immune cells in SCOS than OA samples. Moreover, CD56^bright^ natural killer cell was significantly associated with six hub genes. Enriched hallmark pathways in SCOS had remarkably more upregulated pathways than the downregulated ones. Collectively, we detected DEGs, significant modules, hub genes, upstream TFs and kinases, enriched downstream pathways, and infiltrated immune cells that might be specifically implicated in the pathogenesis of SCOS. These findings provide new insights into the pathogenesis of SCOS and fuel future advances in its theranostics.

## Introduction

Infertility is a major problem for human global health, affecting 15% of couples of reproductive age (1). Male infertility accounts for around half of human infertility and poses a significant challenge to efforts to understand and treat spermatogenesis abnormalities (2). Non-obstructive azoospermia (NOA) has emerged as the most vexing type of male infertility and occurs in 10–15% of infertile men (1). Notably, only a fraction of NOA patients have known etiologies such as Klinefelter syndrome and Y chromosome microdeletion. On the other hand, up to 70% of NOA patients have unknown etiology and are therefore categorized as idiopathic NOA (3).

First described by Del Castillo et al. in 1947 (4), SCOS occurs in 26.3–69.6% of NOA patients (5–7). In seminiferous tubule, spermatogonial stem cells are continuously undergoing self-renewal or differentiating into spermatozoa. These two important trends maintain a balance which is primarily modulated by Sertoli cells. Additionally, Sertoli cells also sustain the blood-testis barrier and function in the testicular microenvironment (8). The imbalance between these two trends disrupts spermatogenesis thoroughly, which in turn leads to the initiation and progression of SCOS. SCOS testes is typically characterized by Sertoli cells instead of spermatogenic cells lining the seminiferous tubules, as well as markedly atrophied seminiferous tubule with thickened tubule walls. In rare cases with SCOS, very few segments of seminiferous tubules are dilated and capable of producing spermatozoa (3). For these small number of cases with SCOS, a few spermatozoa could be acquired intraoperatively, enabling the implementation of intracytoplasmic sperm injection (ICSI). Despite this, the fertility dilemma would be inherited to the next generation even when the success of ICSI is accomplished. Symptomatically, SCOS often manifests as azoospermia, normal virilization, as well as normal to atrophic testis. Besides, elevated follicle-stimulating hormone (FSH) level, normal luteinizing hormone (LH) level, and normal testosterone level are common endocrine manifestations of SCOS patients (9). The etiology of SCOS remains largely unknown, although chemotherapy, radiation, cryptorchidism, and chromosome abnormality have been implicated in the etiology of SCOS (10–12). Furthermore, aberrant expression of NANOS1, SYCP3, PLK4, TEX11, TAF4B, ZMYND15, and HSF2 was reported to be associated with SCOS (13–16).

Bioinformatics analysis based on expression profile data has seen a rise in popularity in recent years, which has been validated as an excellent way to identify underlying mechanisms in a variety of human diseases. In sharp contrast, genome-wide studies in the context of SCOS men are extremely limited. On the other hand, little is known about the molecular mechanisms underlying the pathogenesis of SCOS and there are no clinically validated therapeutic agents for this disease. In doing so, the demand for exploring the mechanistic aspects underlying SCOS for future therapeutic tailoring has taken on increasing urgency. Thus far, there has been a handful of genome-wide studies concerning the pathogenesis of SCOS through research on several cases or one dataset (17, 18). Gratifyingly, a recent study conducted a meta-analysis of three datasets to compare the transcriptome of SCOS men with that of obstructive azoospermia (OA) men (19). In their study, the protein-protein interaction (PPI) network was established but no attempt was made to find important module(s) within the network. Functional enrichment was limited to Gene Ontology (GO) and Kyoto Encyclopedia of Genes and Genomes (KEGG) pathway analyses. Moreover, neither upstream regulatory network construction nor consensus module analysis was performed in their work. To increase the statistical power, our current study included additional datasets that were not included in their study. Based on a comprehensive genomic analysis of six public datasets, we aimed to investigate the putative important genes, critical modules, transcription factors (TFs), kinases, pathways, and infiltrating immune cells implicated in the pathogenesis of SCOS.

## Materials and Methods

### Data Acquisition

The data associated with SCOS and/or OA were achieved from the Gene Expression Omnibus (GEO, https://www.ncbi.nlm.nih.gov/geo/) database portal *via* the keyword “Sertoli cell-only syndrome” or “obstructive azoospermia”. Data inclusion criteria were defined as follows: (a) the organism belonged to Homo sapiens; (b) samples incorporated SCOS and/or OA testicular tissue; and (c) data for SCOS and/or OA testicular tissue samples were intact. Only those datasets that were compliant with all the above-mentioned requirements were enrolled. In view of this, six datasets, namely, GSE45885 (20), GSE4797 (21), GSE6023 (22), GSE21613 (23), GSE9210 (24), and GSE145467 (25) were enrolled for further analysis. In other words, 38 OA testicular samples and 30 SCOS testicular samples were enrolled, and detailed information regarding these six datasets is listed in **Supplementary Table S1**.

### Data Pre-Processing and Differentially Expressed Genes (DEGs) Detection

The software R (https://www.bioconductor.org/) was implemented for data analysis. We imported original data files (^∗^.CEL) of the six datasets through the R package *oligo* (26). Then, we serially performed filtration, background correction, log transformation (base = 2), and normalization of the data. Specifically, we filtered out probes with small variance across samples using the R package *genefilter*. Normalization was conducted using robust multi-chip average (RMA) in the R package *affy*. In addition, we performed batch effect correction by using the ComBat function in the *sva* R package (27). According to the annotation information of the platform, a gene symbol was achieved after transformation of the probe. When one gene symbol exhibited more than one corresponding probe, the mean expression value of these multiple probes was picked out as output. The distribution types of OA and SCOS testicular samples prior to and after clustering and removing outliers were implemented through principal component analysis (PCA). DEGs between OA and SCOS testicular samples were acquired through the R package *limma* (28), with a significance cut-off of false discovery rate (FDR) adjusted *P* value < 0.05 and |log2FC| > 1. Subsequently, the heatmap was generated using the R package *pheatmap* with Euclidean distance and complete linkage clustering method.

### Functional Enrichment

GO terms and KEGG pathway analyses were carried out through the R package *clusterProfiler* (29) for gene functional annotation. GO terms contained biological process (BP), cellular component (CC), and molecular function (MF). Significant enrichment was defined as an adjusted *P* value < 0.05.

### PPI Network Construction

The PPI network was established through the Search Tool for the Retrieval of Interacting Genes (STRING) online database (30), with a significance cut-off of interaction score over 0.9. Then, the Cytoscape software was implemented for a better visualization of PPI network (31). We applied Molecular Complex Detection (MCODE) (32) plugin in Cytoscape for the extraction of densely connected modules from the PPI network. The modules with the top five highest MCODE scores were treated as the hub modules. Also, we selected the Cytoscape plugin CytoHubba (33) to detect key genes from the PPI network. The top 100 genes from each approach of CytoHubba ranking were extracted, followed by the intersection of these genes from all 11 approaches. Here the 11 approaches of CytoHubba ranking were Betweenness, Stress, Radiality, Eccentricity, node connect degree (Degree), density of maximum neighborhood component (DMNC), edge percolated component (EPC), maximal clique centrality (MCC), node connect closeness (Closeness), maximum neighborhood component (MNC), and BottleNeck. All 11 methods of the CytoHubba plugin were sequentially applied. The top 100 genes obtained using each method are listed in **Supplementary Table S2**.

### Consensus Module Analysis

A signed weighted gene co-expression network was established through the R package *WGCNA* (34). The intact gene expression matrix included a total of 9824 genes across OA and SCOS testicular samples. After excluding the genes with the lowest variance (bottom quartile), both expression matrixes of OA and SCOS groups contained 7368 genes. We merged the common genes of the two groups, which left 6580 genes in each group for further analysis. We established the soft threshold power β to construct a scale-free network in the matrix of each group using the pickSoftThreshold function in the R package *WGCNA*. Then, we transformed the resulting Pearson correlation matrix to the adjacency matrix, which was then converted into a topological overlap matrix (TOM). According to the TOM, the corresponding inconsistency was calculated. We subsequently constructed a gene co-expression network separately for each group with the blockwiseModules function in the R package *WGCNA*, followed by the construction of consensus gene co-expression network across these two groups with the blockwiseConsensusModules function in the R package *WGCNA*. Then, we examined the consensus modules formed by the gene co-expression networks through the dynamic tree cut method (35, 36). We subsequently related OA-specific or SCOS-specific modules to the consensus ones. The overlaps of each pair of specific and consensus modules were calculated, and Fisher’s exact test was applied for the assignment of a *P* value to each pairwise overlaps.

### Upstream Regulator Network Construction

The eXpression2Kinases (X2K) (https://amp.pharm.mssm.edu/X2K/) is utilized to compute the regulatory correlations among TFs, kinases, and intermediate proteins according to hypergeometric *P* value (37). Here, X2K was applied for the construction of the upstream regulator network that regulated the downregulated genes.

### Identification of Immune Cells Infiltrated in OA and SCOS Testicular Tissue Samples

We implemented single-sample GSEA (ssGSEA) using the R package *GSVA* to assess the enrichment score of each testicular tissue sample (38). Correspondingly, gene expression values of OA and SCOS testicular tissue samples and the metagenes of 28 kinds of immune cells were utilized (39). The normalized enrichment score (NES) achieved through ssGSEA was treated as the immune‐infiltrating value for the enrichment analysis of 28 immune cells. We then calculated the association between each hub gene and the immune cell.

### Gene Set Variation Analysis (GSVA) and Gene Set Enrichment Analysis (GSEA)

To further refine enrichment analysis, the dysregulated hallmark pathways in SCOS were determined *via* two different approaches. First, GSVA was conducted through the R package *GSVA* (38). Specifically, we obtained the gene set “h.all.v7.1.symbols.gmt” from the Molecular Signatures Database (https://www.gsea-msigdb.org/gsea/index.jsp). Second, GSEA was conducted using the R package *fgsea*. Here, we summarized the three most significantly enriched hallmark pathways using a gseaplot and presented the enriched genes for the four most significantly enriched hallmark pathways through a cnetplot, respectively. Finally, we intersected the hallmark pathways acquired *via* GSVA with those obtained from GSEA.

## Results

### DEGs Identification

**Figure 1:**
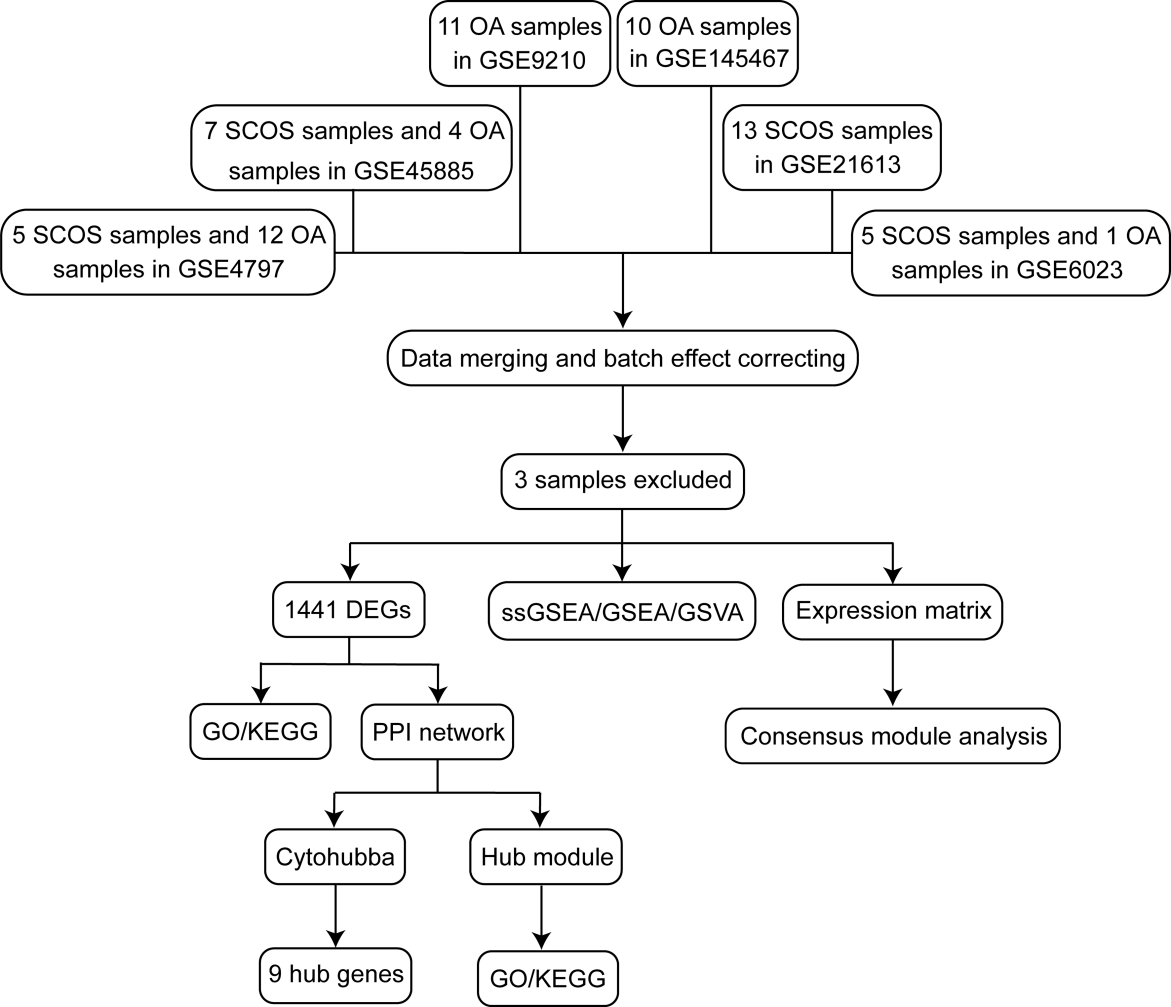
Flowchart of the study design. DEGs, differentially expressed genes; GO, gene ontology; GSEA, gene set enrichment analysis; GSVA, gene set variation analysis; KEGG, Kyoto Encyclopedia of Genes and Genomes; OA, obstructive azoospermia; PPI, protein-protein interaction; SCOS, Sertoli cell-only syndrome.

**Figure 2:**
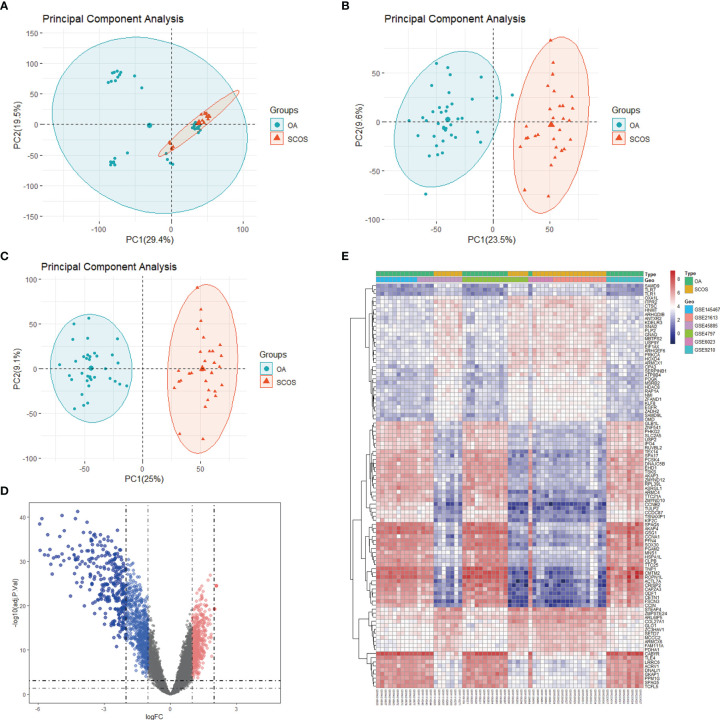
Data pre-processing and DEGs identification. Principal component analysis (PCA) depicting the whole profile of the six datasets **(A)** prior to and **(B)** after batch correction and data fusion. **(C)** PCA after outlier removal. **(D)** A volcano plot depicting the distribution of DEGs between SCOS and OA samples from the six datasets. Red, blue, and gray dots represent gene expression levels corresponding to upregulated, downregulated, and insignificant expression. **(E)** A heatmap depicting the top 50 upregulated and top 50 downregulated DEGs. Red and blue dots represent gene expression levels corresponding to upregulated and downregulated expression. DEGs: differentially expressed genes; OA, obstructive azoospermia; SCOS, Sertoli cell-only syndrome.

A detailed flowchart depicting this study is shown in **Figure 1**. First, a combination of six publicly available microarray gene expression datasets with SCOS and/or OA samples were utilized. The expression values of these samples prior to and after normalization are shown as bar graphs (**Supplementary Figures S1A, B**). PCA was implemented for the visualization of the distribution of these samples prior to and after correcting batch effect (**Figures 2A, B**). In addition, PCA was also performed before and after cleaning three outlier samples (GSM108235, GSM233018, and GSM233026) (**Figures 2B, C**). In doing so, a total of 35 OA and 30 SCOS testicular tissue samples remained. After filtration, we detected 1441 DEGs, including 508 upregulated genes and 933 downregulated genes. Also, the ratio of the number of downregulated genes to that of the upregulated ones was 1.84:1. All DEGs were visualized using a volcano plot (**Figure 2D**), and it could be seen that there are more downregulated genes with extremely significant adjusted *P* values and absolute log2FC in comparison with the upregulated ones. Furthermore, the top 50 upregulated concurrent with the top 50 downregulated DEGs were shown using a heatmap (**Figure 2E**).

### Enrichment Analyses

**Figure 3:**
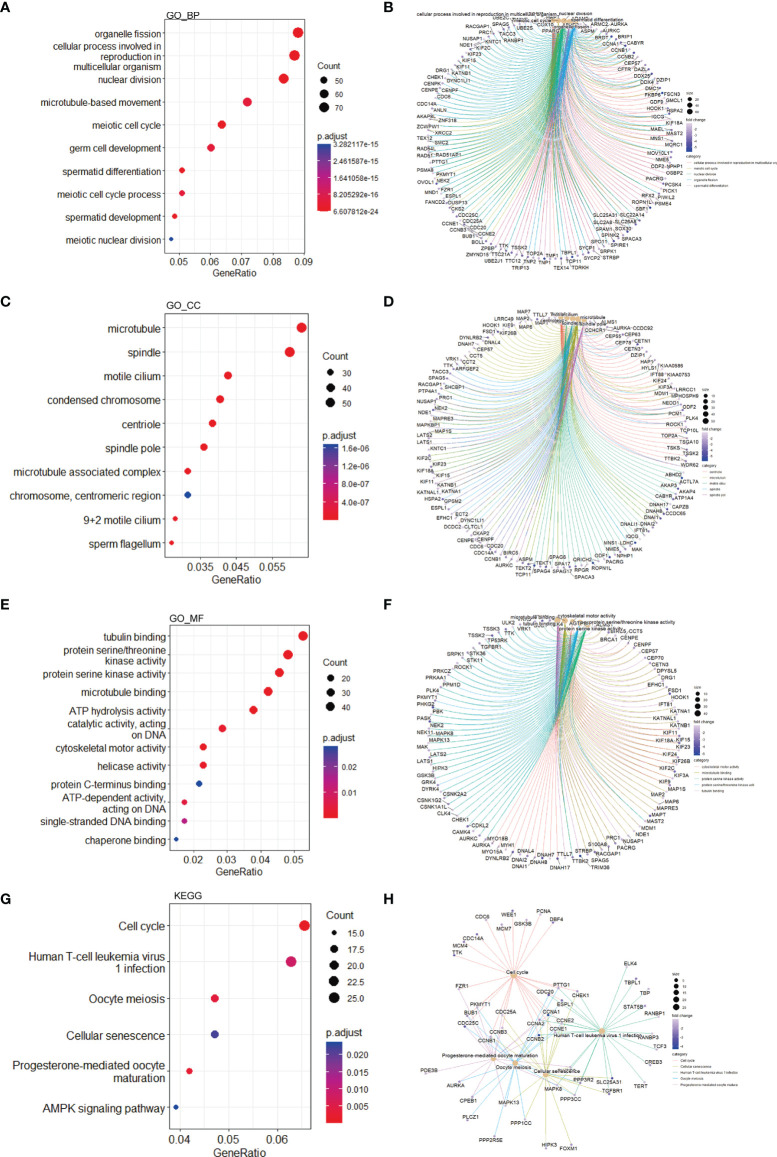
Functional enrichment based on downregulated DEGs. GO-BP analysis for the downregulated DEGs showing the significant terms through **(A)** a bubble plot and specific genes associated with these terms through **(B)** a cnetplot. GO-CC analysis for the downregulated DEGs showing the significant terms through **(C)** a bubble plot and specific genes associated with these terms through **(D)** a cnetplot. GO-MF analysis for the downregulated DEGs showing the significant terms through **(E)** a bubble plot and specific genes associated with these terms through **(F)** a cnetplot. KEGG analysis for the downregulated DEGs indicating the enriched pathways through **(G)** a bubble plot and specific genes associated with these pathways through **(H)** a cnetplot. BP, biological process; CC, cellular component; DEGs, differentially expressed gene; GO, Gene Ontology; KEGG, Kyoto Encyclopedia of Genes and Genomes; MF, molecular function.

**Figure 4:**
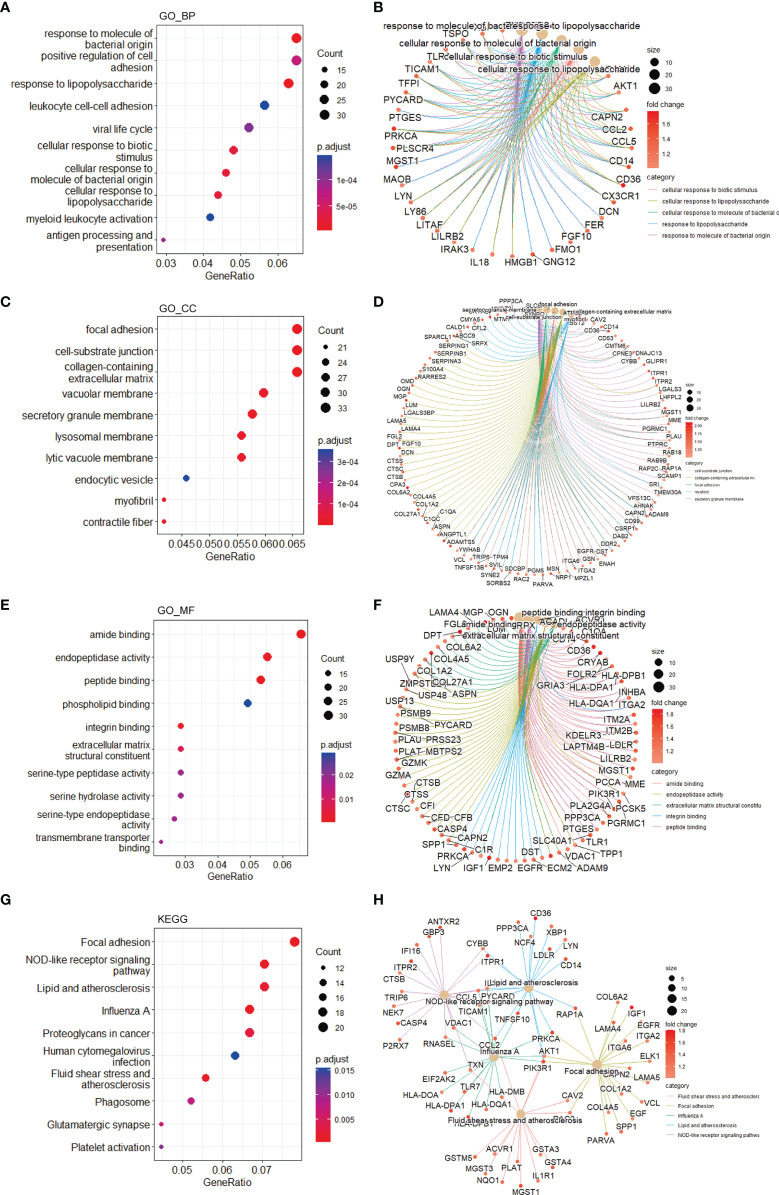
Functional enrichment based on upregulated DEGs. GO-BP analysis for the upregulated DEGs showing the significant terms through **(A)** a bubble plot and specific genes associated with these terms through **(B)** a cnetplot. GO-CC analysis for the upregulated DEGs showing the significant terms through **(C)** a bubble plot and specific genes associated with these terms through **(D)** a cnetplot. GO-MF analysis for the upregulated DEGs showing the significant terms through **(E)** a bubble plot and specific genes associated with these terms through **(F)** a cnetplot. KEGG analysis for the upregulated DEGs indicating the enriched pathways through **(G)** a bubble plot and specific genes associated with these pathways **(H)** a cnetplot. BP, biological process; CC, cellular component; DEGs, differentially expressed gene; GO, Gene Ontology; KEGG, Kyoto Encyclopedia of Genes and Genomes; MF, molecular function.

To investigate the biological functions of the DEGs, we performed GO terms and KEGG pathways enrichment analyses. In accordance with the downregulated DEGs, enriched GO terms included organelle fission, a cellular process involved in reproduction in multicellular organisms, nuclear division, etc. (**Figures 3A, C, E**). Moreover, the enriched genes for specific GO terms are plotted through cnetplots (**Figures 3B, D, F**). In addition to GO terms, enriched KEGG pathways contained cell cycle and pathways related to reproduction (**Figure 3G**). Furthermore, the enriched genes for specific KEGG pathways are plotted in **Figure 3H**. As for the upregulated DEGs, many enriched GO terms were related to inflammation, such as response to molecule of bacterial origin, positive regulation of cell adhesion, and response to lipopolysaccharide (**Figures 4A, C, E**), while enriched KEGG pathways included focal adhesion, NOD-like receptor signaling pathway, lipid, and atherosclerosis, etc. (**Figure 4G**). In addition, the enriched upregulated genes for specific GO terms and KEGG pathways are plotted through cnetplots (**Figures 4B, D, F, H**).

### PPI Network and Its Hub Modules

**Figure 5:**
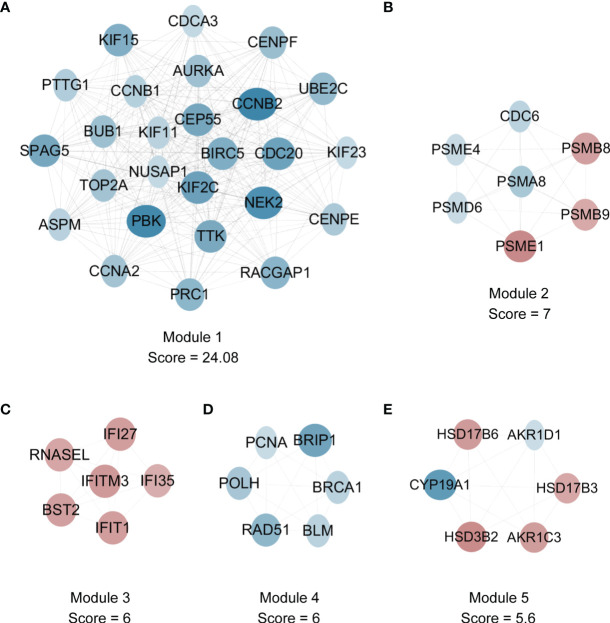
The detection of hub modules in PPI network. **(A)** There are 26 nodes and 301 edges in module 1. **(B)** There are 7 nodes and 21 edges in module 2. **(C)** There are 6 nodes and 15 edges in module 3. **(D)** There are 6 nodes and 15 edges in module 4. **(E)** There are 6 nodes and 14 edges in module 5. Red and blue nodes represent gene expression levels corresponding to upregulated and downregulated expression. DEGs, differentially expressed genes; PPI, protein-protein interaction.

For understanding the interactions among the DEGs, we constructed the PPI network, which included 1437 nodes and 3581 edges (**Supplementary Figure S2**). Among the hub modules, there were 26 nodes and 301 edges in module 1, whose MCODE score was the highest (24.08). Notably, all genes within module 1 (**Figure 5A**) and module 4 (**Figure 5D**) belonged to the downregulated DEGs. As for module 2 (**Figure 5B**) and module 5 (**Figure 5E**), there were both upregulated and downregulated genes. In addition, all genes within module 3 (**Figure 5C**) belonged to the upregulated DEGs.

Based on all genes within module 1, subsequent GO and KEGG enrichment analyses were conducted. It was found that GO-BP terms consisted of nuclear division, organelle fission, chromosome segregation, etc. (**Supplementary Figure S3A**); GO-CC terms included spindle, microtubule, midbody, condensed chromosome, etc. (**Supplementary Figure S3C**); GO-MF terms contained microtubule binding, tubulin binding, cytoskeletal motor activity, etc. (**Supplementary Figure S3E**); enriched KEGG pathways were comprised of cell cycle, oocyte meiosis, etc. (**Supplementary Figure S3G**). The enriched genes for specific GO terms and enriched KEGG pathways are shown through heatmaps (**Supplementary Figures S3B, D, F, H**). Overall, the GO terms and KEGG pathways enriched by genes in module 1 were related to cell cycle related events, which were very similar to those enriched by all downregulated genes.

**Figure 6:**
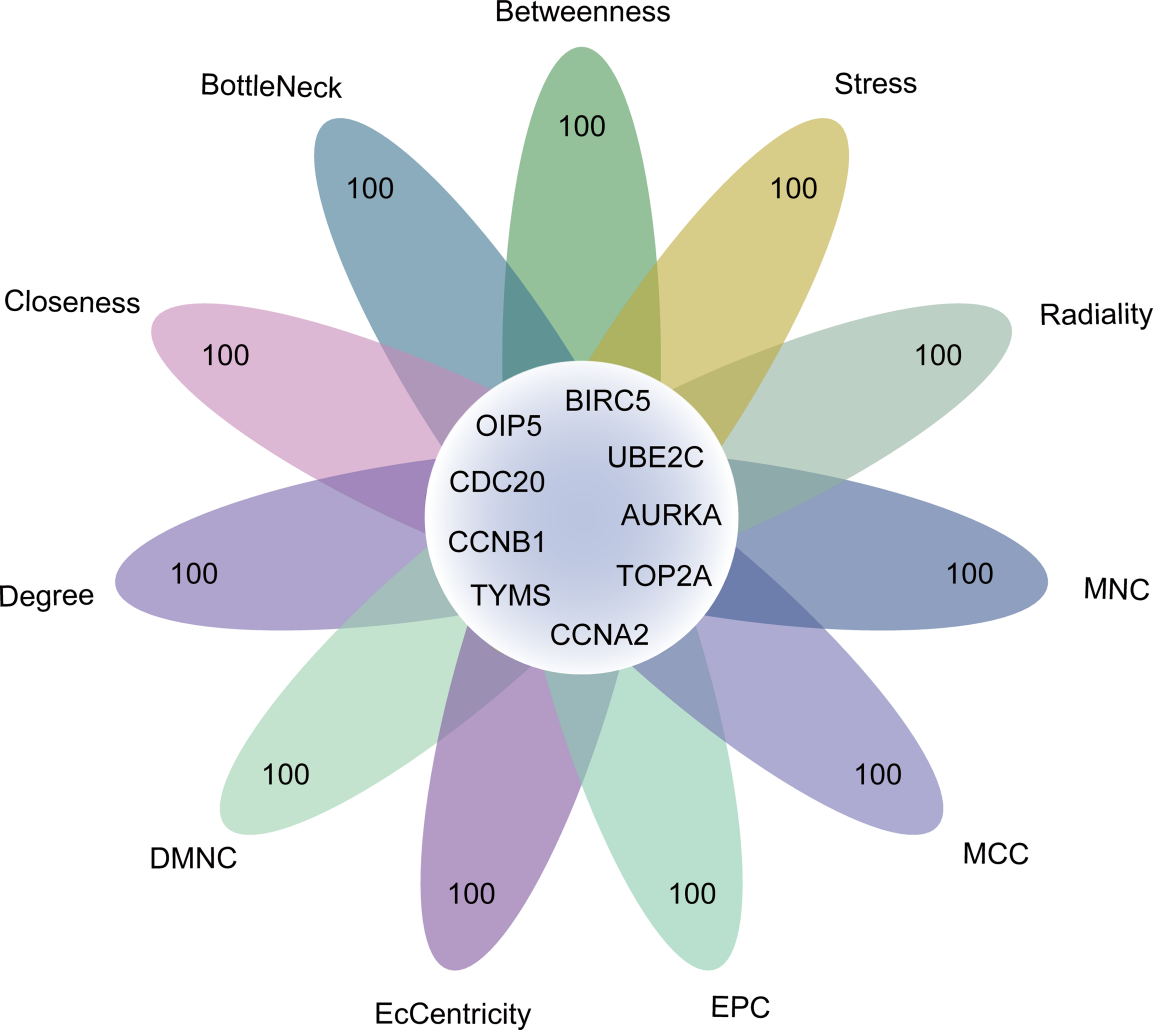
The identification of 9 genes through intersecting genes from 11 ranking methods in the PPI network. Degree, node connect degree; DMNC, density of maximum neighborhood component; EPC, edge percolated component; MCC, maximal clique centrality; MNC, maximum neighborhood component; Closeness, node connect closeness; PPI, protein-protein interaction.

To further elucidate highly connected genes in the PPI network, we applied 11 approaches. Based on the intersection of the top 100 genes through all 11 approaches, 9 hub genes were obtained, including CCNB1, CCNA2, BIRC5, TYMS, UBE2C, OIP5, AURKA, CDC20, and TOP2A (**Figure 6**). Interestingly, all these intersected genes, without exception, were found to be downregulated.

### Consensus and SCOS-Specific Modules

**Figure 7:**
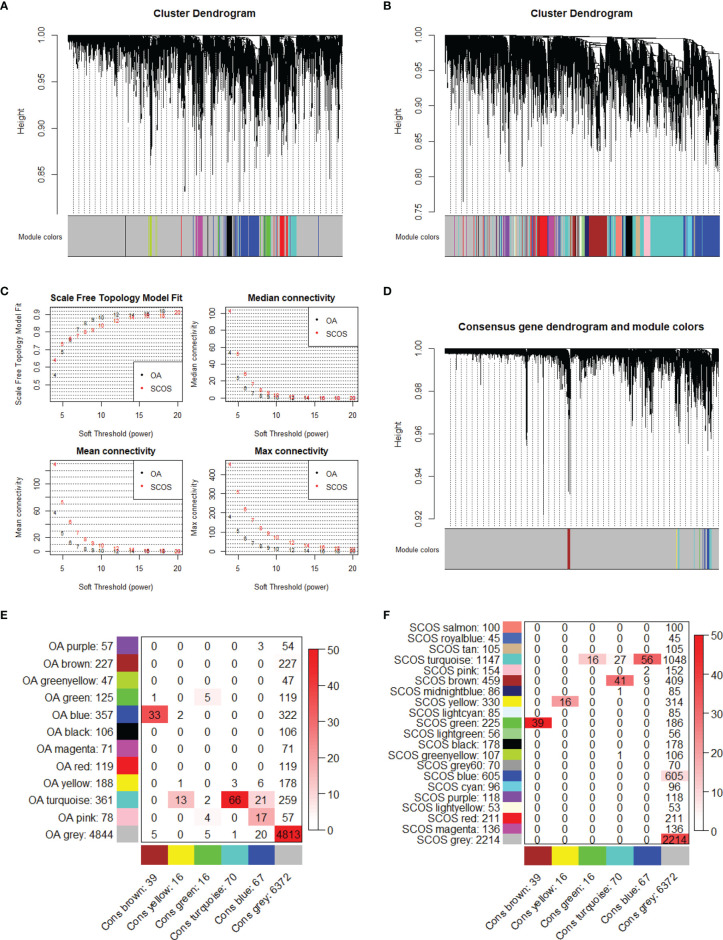
Network construction in each group and consensus module analysis. Cluster dendrograms of **(A)** OA and **(B)** SCOS modules. **(C)** Soft threshold and network connectivity. The networks of OA and SCOS groups formed a scale-free network, in which median, mean, or max connectivity indicated node connectivity. **(D)** Cluster dendrogram of the OA-SCOS consensus modules. **(E)** Correspondence of OA-specific modules and the OA-SCOS consensus modules. **(F)** Correspondence of SCOS-specific modules and the OA-SCOS consensus modules. The number in each small square means gene counts. A stronger red color suggests more significant overlap. OA, obstructive azoospermia; SCOS, Sertoli cell-only syndrome.

After removal of the genes with the bottom quartile and merging common genes within OA and SCOS groups, up to 6580 genes was utilized for the construction of OA-specific, SCOS-specific, and consensus gene co-expression networks. During sample clustering, no sample was taken as the outlier (**Supplementary Figure S4**). Further, β = 12 was treated as the optimal soft threshold parameter to ensure scale-free network of both OA and SCOS groups (**Supplementary Figure S5** and **Figure 7C**). Subsequently, a gene co-expression network was built separately for each group, and we obtained 12 and 20 gene co-expression modules for OA and SCOS groups, respectively (**Figures 7A, B**). Then, six consensus modules within the consensus gene co-expression network were established across these two groups (**Figure 7D**). The correspondence of OA-specific/SCOS-specific modules and the consensus modules was also identified (**Figures 7E, F**). Notably, it could be seen that most SCOS-specific modules did not have a consensus counterpart, and this was not unexpected due to the sharp difference of gene expression profiles between the OA and SCOS groups. As for the SCOS-specific modules, the green module containing 225 genes showed the most significant overlap with the consensus brown module. Also, it may not be a coincidence that, of the 9 hub genes obtained from the PPI network, 6 also belonged to the genes within the SCOS-specific green module.

Based on the clustering dendrograms, we detected some level of preservation of consensus modules within the OA and SCOS groups (**Supplementary Figures S6A, B**), and the heatmaps varied slightly from each other (**Supplementary Figures S6C, D**). Also, the adjacency heatmap and the high-density value verified the preservation of consensus modules (**Supplementary Figures S6E, F**).

To understand the biological functions of the consensus and SCOS-specific modules, we performed GO terms and KEGG pathways enrichment analyses. None of the consensus modules showed significant enrichment. In addition, based on the union of genes in all consensus modules (except the gray module), we did not obtain any enrichment of statistical significance. When it comes to the SCOS-specific modules, we detected significant enrichment for green, red, cyan, brown, lightcyan, greenyellow, salmon, and gray60 modules. In detail, the enrichment for the SCOS-specific green module yielded various cell cycle related GO terms and KEGG pathway, such as tubulin binding and microtubule binding (**Supplementary Figure S7**). Enriched GO terms for the SCOS-specific red module included amide binding, growth factor binding, and extracellular matrix structural constituent, while enriched GO terms for the SCOS-specific brown module contained actin binding, phosphoric ester hydrolase activity, calcium-dependent protein binding, and semaphorin receptor activity. Moreover, cytokine-cytokine receptor interaction was identified as a significantly enriched KEGG pathway for the SCOS-specific gray60 module (**Supplementary Figure S8**). On the other hand, no significant enrichment was observed for royalblue, tan, turquoise, pink, midnightblue, yellow, lightgreen, black, blue, purple, lightyellow, and magenta SCOS-specific modules.

### Upstream Regulatory Network

**Figure 8:**
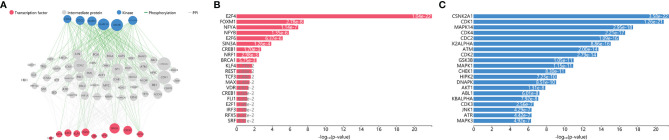
Upstream regulatory network prediction according to the downregulated DEGs. **(A)** Regulatory network diagram according to the prediction of the downregulated DEGs. Nodes’ size is scaled proportional to the corresponding degree. **(B)** Kinases and **(C)** transcription factors according to the predictions of the downregulated DEGs. DEGs, differentially expressed genes.

Considering the imbalance between the upregulated and downregulated genes, the downregulated genes were utilized to predict the upstream regulatory network in the pathogenesis of SCOS. Specifically, we predicted TFs, kinases, and intermediate proteins (**Figure 8A**). A total of 63 intermediate proteins with 658 edges were linked to the TFs and kinases. The predicted upstream TFs included E2F4, FOXM1, NFYA, NFYB, E2F6, SIN3A, CREB1, NRF1, BRCA1, KLF4, etc. (**Figure 8B**, **Supplementary Table S3**). Notably, NFYB and E2F6 were predicted to target more than 200 genes, while E2F4, NFYA, NRF1, and BRCA1 were predicted to target over 100 genes. Besides, the most significantly correlated kinases included CSNK2A1, CDK1, MAPK14, CDK4, CDC2, CK2ALPHA, ATM, CDK2, GSK3B, MAPK1, etc. (**Figure 8C**; **Supplementary Table S4**). It was found that a group of CDK and MAPK like protein kinases were highly associated with the downregulated genes.

### Immune Cells Infiltration and Dysregulated Hallmark Pathways

**Figure 9:**
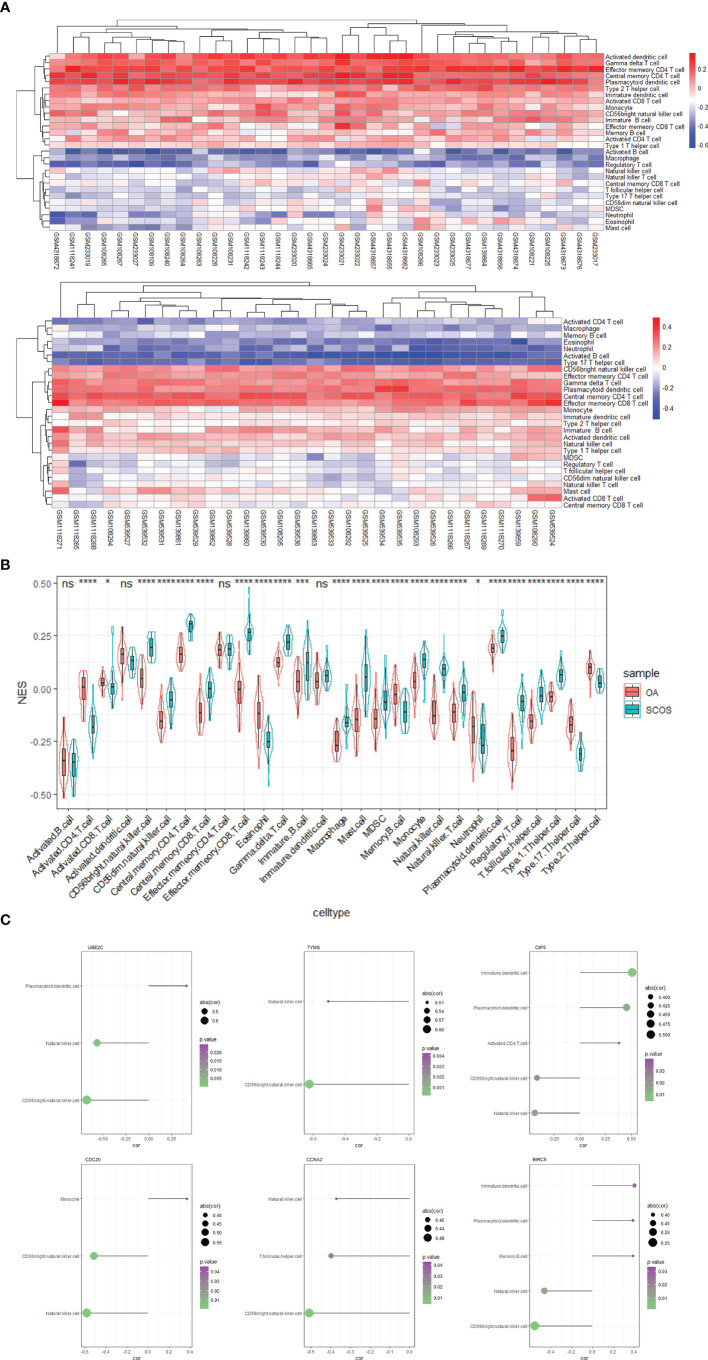
The immune cells and OA/SCOS testicular tissue samples. **(A)** The heatmap summarizing the correlations between OA/SCOS testicular tissue samples and immune cells. The upper heatmap corresponds to the OA samples, while the lower heatmap corresponds to the SCOS samples. **(B)** Bar graph nested by violin plot exhibiting different levels of infiltrating immune cells between OA and SCOS testicular tissue samples. **(C)** The forest plots showing the significant associations between hub genes and immune cells including CD56^bright^ natural killer cell. NES, normalized enrichment score; OA, obstructive azoospermia; SCOS, Sertoli cell-only syndrome. *P value < 0.05; ***P value < 0.001; ****P value < 0.0001; ns, no significance.

Given that many of GO terms and KEGG pathways enriched by the upregulated DEGs were related to inflammation and infection (**Figure 4**), we speculated that immune cell infiltration might play an essential part in the pathogenesis of SCOS. Thus, we investigated the associations between OA/SCOS testicular tissue samples and infiltrated immune cells. As depicted in **Figure 9A**, the overall infiltration levels of immune cells varied greatly between OA and SCOS groups. Subsequently, levels of each infiltrated immune cell were compared between SCOS and OA testicular tissue samples. For most immune cells, a significantly higher infiltrated degree was observed in the SCOS group than the OA group (**Figure 9B**), which echoed the enrichment results according to the upregulated genes (**Figure 4**). Furthermore, the significant correlations between each hub gene and the corresponding immune cells were also detected (**Figure 9C**; **Supplementary Figure S9**). It should be noted that, among the nine hub genes, natural killer (NK) cell and CD56^bright^ NK cell were significantly associated with 7 and 6 hub genes, respectively.

**Figure 10:**
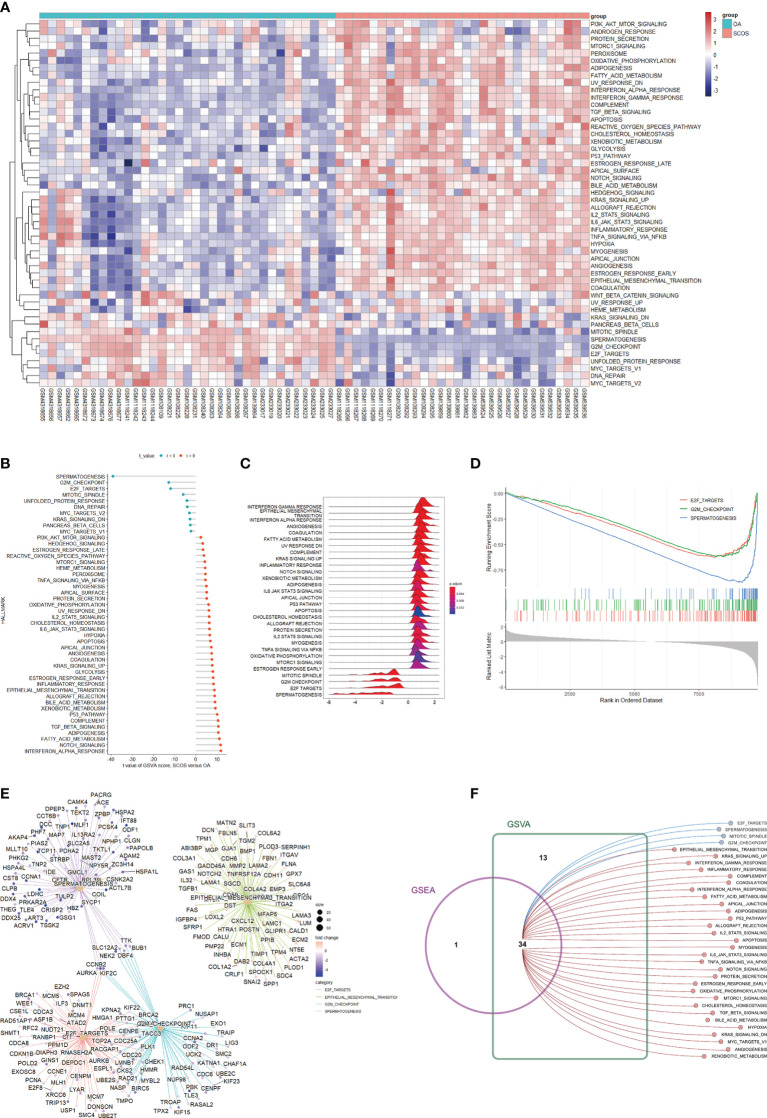
GSEA and GSVA uncovering enriched hallmark pathways in SCOS. **(A)** The heatmap indicating specific hallmark pathways and testicular tissue samples determined by GSVA enrichment. **(B)** The lollipop plot showing dysregulated hallmark pathways in SCOS acquired by GSVA enrichment. **(C)** The ridgeplot visualizing the aberrantly expressed hallmark pathways in SCOS determined by GSEA enrichment. **(D)** The gseaplot identifying the three most significantly enriched hallmark pathways between SCOS and OA testicular tissue samples. **(E)** The cnetplot showing specific genes enriched in different aberrantly expressed hallmark pathways. **(F)** GSEA and GSVA for significantly enriched hallmark pathways in SCOS. GSEA, gene set enrichment analysis; GSVA, gene set variation analysis; OA, obstructive azoospermia; SCOS, Sertoli cell-only syndrome.

To unveil enriched hallmark pathways in SCOS, we first focused on the correlation of specific hallmark pathway expression and each testicular tissue sample. Taken as a whole, **Figure 10A** gives us an intuitive impression that most hallmark pathways were significantly upregulated in the SCOS group while an opposite trend was observed for the OA group. The dysregulated hallmark pathways in SCOS were determined *via* two different approaches, which are shown through a lollipop plot (**Figure 10B**) and a ridgeplot (**Figure 10C**), respectively. As for enrichment results using the second method, the three most significantly enriched hallmark pathways in SCOS, including E2F targets, G2M checkpoint, and spermatogenesis, are shown using a gseaplot (**Figure 10D**). In addition, the enriched genes for the four most significantly enriched hallmark pathways are plotted through a cnetplot (**Figure 10E**). We then intersected the hallmark pathways predicted *via* these two methods, ultimately resulting in 30 upregulated and 4 downregulated hallmark pathways (**Figure 10F**) and corroborating the impression given by **Figure 10A**.

## Discussion

The histopathological types of NOA include hypospermatogenesis (HS), maturation arrest (MA), and SCOS (40). Different histopathological types of NOA exhibited different gene expression profiles (21). In addition, Dorosh et al. examined the expression levels of spermatogenesis-related genes (MND1, SPATA22, GAPDHS, and ACR) in testicular tissues from NOA men, and found that gene expression was significantly decreased for SPATA22 and GAPDHS in the SCOS group compared with that in the HS and MA groups (41). Moreover, the extent of variation in the proportions of NOA samples with different pathological types ranged widely in different studies (5–7). If gene expression profiles of NOA are directly compared with those of OA, distinct proportions of NOA samples with different pathological types may intrinsically lead to different results. Therefore, when the pathological types of NOA testicular tissue samples are already known, it may be too general and slightly inappropriate to directly compare gene expression profiles between NOA and OA groups. Given that SCOS is the most severe and common pathological type of NOA (42), this study only included testicular tissue samples in men with SCOS or OA to provide a precise description of aberrant gene expression profiling in men with SCOS.

Among the 9 hub genes identified in this study, CCNB1, CCNA2, and AURKA have been previously reported as hub genes associated with SCOS in men (19). Of the remaining 6 hub genes, BIRC5 belongs to the inhibitor-of-apoptosis proteins (IAPs) family. The function of BIRC5 in the regulation of apoptosis and mitosis has been well established. To be specific, the activities of caspases can be suppressed *via* binding to BIRC5. Also, BIRC5 can bind with CDCA8, AURKB, and INCENP to form a complex, which in turn affects microtubule dynamics during the G2/M phase (43). As for men with oligo-astheno-teratozoospermia, it was reported that both concentration and motility of spermatozoa were positively associated with the expression levels of BIRC5 in seminal plasma. On the other hand, BIRC5 was not detectable in seminal plasma from NOA men with unsuccessful sperm retrieval (44), which thus corroborated the downregulation of BIRC5 in SCOS testicular tissue samples from our current study. TYMS, primarily enriched at the G1 to S phase transition, enables the catalyzation of methylating deoxyuridylate to deoxythymidylate with the help of 5,10-methylenetetrahydrofolate, thereby maintaining the pool of thymidine-5-prime monophosphate as well as protecting DNA replication and repair. Animal experiments demonstrated that the abnormal expression of TYMS might be implicated in the failure of post-implantation development for the embryo (45). During the metaphase-anaphase transition, CDC20 is a mitotic activator of anaphase-promoting complex/cyclosome (APC/C) and can successively disrupt CCNB1 and PTTG1 for the initiation of sister-chromatid separation. It has been described that the missense mutation of CDC20 (CDC20 R383C) was correlated with the pathogenesis of NOA in men (46). In female infertility, five types of mutations in CDC20 were reported to be associated with oocyte maturation arrest in female patients (47). UBE2C serves as a member of ubiquitin-conjugating enzyme (E2) family and plays an essential part in mitosis in human cells. The cell cycle can be accelerated by UBE2C *via* its interaction with APC/C. UBE2C is involved in the dissociation of the MAD2-CDC20 complex through ubiquitylating CDC20, resulting in the impairment of spindle assembly checkpoint (48). Much progress has been made in the role of UBE2C in the field of female reproduction. In porcine oocytes, UBE2C coordinated with UBE2S promoted the escape from MII arrest through ubiquitylating certain factors (49). First detected in a yeast two-hybrid system (50), OIP5 is enriched in the centromere and plays an important role in the mitosis through the interaction with the Holliday junction recognition protein and the recruitment of centrosome-associated protein A (51). TOP2A is a member of the topoisomerase II family responsible for topologic states of DNA during the course of transcription and replication. TOP2A and small ubiquitin-like modifier proteins were reported to involve regulating dynamics of meiotic chromosome in germ cells (52). Several previous studies (53–56) integrated published datasets to compare the transcriptomes of NOA testicular tissue samples as a whole with OA samples, and also identified some of the aforementioned hub genes to be highly relevant to the presence of NOA in their studies. To be specific, Kui et al. reported UBE2C, CDC20, and TOP2A to be essential for NOA pathogenesis in their work (53), while Cao et al. identified TYMS, OIP5, and BIRC5 as genes highly correlated with NOA (55). It could be explained by a high proportion of SCOS samples in the NOA group, as well as many hub genes observed in these previous studies. For instance, more than 60 hub genes were achieved in the study done by Cao et al. (55).

Our results demonstrated many highly associated TFs in SCOS. Among these TFs, E2F4 belongs to the E2F family that is implicated in the in-and-out events of the cell cycle. A report has suggested that combined mutation of E2F4 and E2F5 could cause multiciliated cell deficiency in the efferent ducts, as well as dilation of the seminiferous tubules and rete testis in mice (57). FOXM1 serves as a transcriptional activator and is implicated in cell proliferation. During the reprogramming of spermatogonial stem cells (SSCs) to multipotent SSCs, FOXM1 was previously found to be significantly correlated with three pluripotency-related processes during the late stages (58). NFYA, NFYB, and NFYC are the three subunits of a highly conserved trimeric complex that has high affinity for DNA binding. It was reported that the open chromatin of human SSCs was strongly enriched in binding sites of critical TFs including NFYA and NFYB (59). CREB1 is a member of the leucine zipper family responsible for DNA binding. In HEK293 and GC1-spg cells, CREB1 could upregulate CATSPER1, which plays critical roles in flagellum hyperactivation of the spermatozoa (60). Gene deletion of SIN3A in the Sertoli cells was reported to lead to the defect of undifferentiated spermatogonia and the downregulation of stem cell-associated markers in fetal mice (61).

In addition to the predicted TFs, we identified cyclin-dependent kinases (CDKs) as the most significantly enriched kinases in this study. Similarly, previous reports also showed the downregulation of CDKs in NOA (62). CDKs belong to serine/threonine protein kinases that are appreciated as playing critical roles in cell-cycle control. CDK/cyclin complexes are formed by the binding of CDK to a specific cyclin subunit and affect cell proliferation during cell cycle progression. Specifically, the CDK4/cyclin D complex could promote growth and play a key role during G1-to-S phase transition. CDK2/cyclin E complex initiates DNA synthesis at the initial part of the S phase, while CDK1/cyclin B complex is responsible for entering mitosis, disassembling nuclear envelope, and separating centrosomes (63). Furthermore, MAPKs also serve as the significantly correlated kinases in this study. MAPKs are key components of signaling pathways that regulate the proliferation and apoptosis of cells. MAPK1 and central elements correlated with its activation were reported to be present in mammalian spermatozoa (64). MAPK14 negatively regulates cell cycle progression at the G1/S and G2/M transitions through downregulating cyclins and upregulating CDK inhibitors. Also, MAPK14 activity is correlated with inducing apoptosis through cellular stress (65). GSK3B is one of the two isoforms of GSK3 that encodes serine/threonine kinase and negatively regulates glucose homeostasis. A previous study revealed that GSK3A, the other isoform of GSK3, was necessary for the regulation of progressive motility of human spermatozoa (66). CSNK2A1 is also a serine/threonine protein kinase. In male mice, loss of CSNK2A1 expression inhibited the phosphorylation of nuclear proteins in spermatids through nuclear envelope protrusion and chromatin dysregulation (67). Echoed with the findings during the prediction of protein kinases, our enrichment results based on downregulated genes also identified serine/threonine kinase activity as one of the top enriched GO terms (**Figure 3E**). Furthermore, protein serine kinase activity was also identified as the most significantly enriched GO term for the SCOS-specific green module in this study (**Supplementary Figure S7**).

An infective and/or inflammatory condition of the human testis is thought to be one of the most important etiologies of male infertility. A previous study revealed that the spermatogenic dysfunction was directly associated with immune response (68). In addition, overexpressed immune response was perhaps the primary commonality of spermatogenic arrest, oligospermia, and teratospermia (69). Similarly, our results demonstrated that a variety of inflammatory signaling pathways, such as IL2 STAT5 signaling and IL6 JAK STAT3 signaling, were upregulated in SCOS compared with OA (**Figure 10F**). Immune cells, such as mast cells or macrophages, exist in the normal human testis. The previous notion that excessive immune cells could play a significant role in human testicular diseases (70) is supported by our results. This study refined the spermatogenic dysfunction to SCOS in men and found that SCOS testicular tissue samples had significantly higher levels of mast cells and macrophages in comparison with OA samples (**Figure 9B**). Additional mast cells were found to be correlated with acute testicular inflammation (71). Furthermore, abnormal spermatogenic testis exhibited significantly more B lymphocytes, T lymphocytes, macrophages, and mast cells in comparison with the normal spermatogenic testis (68). A report has suggested that macrophages were increasingly present in NOA men, and inhibitory paracrine function of macrophages on Leydig cells may partially lead to the deficit of steroidogenesis and testosterone within the testis (72). More recently, one report showed that NOA testis tissue had significantly elevated immune score and more M1 and M2 macrophages in comparison with the normal testis, which was appreciated as the evidence for the vital role of macrophage polarization in NOA development (73). NK cells are traditionally divided into two subsets (CD56^bright^ and CD56^dim^) in humans. Tissue-resident CD56^bright^ NK cells have been identified in the lymphoid tissues and the liver, as well as the uterus. In the field of male infertility, a previous study reported that CD56^bright^ NK cells in semen were significantly increased for men with oligoasthenospermic or OA in comparison with the normospermic men (74). Our results observed a significantly higher infiltrated degree of CD56^bright^ NK cells in the SCOS group than the OA group, and CD56^bright^ NK cells were significantly associated with most hub genes. It would be interesting to further investigate the function of CD56^bright^ NK cells in testis and address the mechanism underlying the raised infiltrated levels of CD56^bright^ NK cells in SCOS testis. The change of the bacterial microbiome in testicular tissues from SCOS patients was reported to be similar to that in the gut of elderly subjects, which represented a higher level of inflammation (75). Consistent with the literature, our data found that response to molecule of bacterial origin was upregulated in the SCOS group compared with the OA group.

There were limitations of the present study. We did not accomplish experimental verification of the hub genes in SCOS. In addition, clinical factors were not taken into consideration during data analysis due to the lack of clinical information in the datasets.

In conclusion, we detected the DEGs, PPI and WGCNA modules, hub genes, enriched pathways, upstream TFs and kinases, and infiltrated immune cells that might be specifically implicated in the pathogenesis of SCOS. These findings provide new insights into understanding the pathogenesis of SCOS and fuel future advances in the theranostics of this disease.

## Data Availability Statement

The datasets presented in this study can be found in online repository. The names of the repository and accession number can be found in **Supplementary Table S1**.

## Ethics Statement

This study design was reviewed and approved by the medical research ethics committee of The First Affiliated Hospital of Nanjing Medical University. The ethics committee waived the requirement of written informed consent for participation.

## Author Contributions

Conception and design: NS and TC; Administrative support: ZW, XG, and JX; Data analysis and visualization: TC, YW, and LT; Manuscript writing: TC, LT, and YW; Final approval of the manuscript: All authors.

## Funding

This work was supported by the National Natural Science Foundation of China (81871151 and 82071638) and the “333” Project of Jiangsu Province (BRA2018084), and Jiangsu Funding Program for Excellent Postdoctoral Talent.

## Conflict of Interest

The authors declare that the research was conducted in the absence of any commercial or financial relationships that could be construed as a potential conflict of interest.

## Publisher’s Note

All claims expressed in this article are solely those of the authors and do not necessarily represent those of their affiliated organizations, or those of the publisher, the editors and the reviewers. Any product that may be evaluated in this article, or claim that may be made by its manufacturer, is not guaranteed or endorsed by the publisher.

## Supplementary Material

Supplementary Figure 1 Bar graphs depicting expression values of SCOS and OA testicular tissue samples **(A)** prior to and **(B)** after normalization. OA, obstructive azoospermia; SCOS, Sertoli cell-only syndrome.

Supplementary Figure 2 PPI network of DEGs. Red and blue dots represent gene expression levels corresponding to upregulated and downregulated expression. Node size varies scaled proportional to the connectivity of the node and the edge indicates direct correlation. DEGs: differentially expressed genes; PPI: protein-protein interaction.

Supplementary Figure 3 Functional enrichment of genes within module 1. GO-BP analysis for genes in module 1 showing the significant terms through **(A)** a bubble plot and specific genes associated with these terms through **(B)** a heatmap. GO-CC analysis for the genes in module 1 showing the significant terms through **(C)** a bubble plot and specific genes associated with these terms through **(D)** a heatmap. GO-MF analysis for the genes in module 1 showing the significant terms through **(E)** a bubble plot and specific genes associated with these terms through **(F)** a heatmap. KEGG function pathway analysis of genes in module 1 indicating the enriched pathways through **(G)** a bubble plot and specific genes associated with these function pathways through **(H)** a heatmap. BP: biological process; CC: cellular component; DEGs: differentially expressed genes; GO: Gene Ontology; KEGG: Kyoto Encyclopedia of Genes and Genomes; MF: molecular function.

Supplementary Figure 4 Hierarchical cluster analysis of samples in the OA and SCOS testicular tissue samples. OA, obstructive azoospermia; SCOS, Sertoli cell-only syndrome.

Supplementary Figure 5 After network topology analysis for soft-threshold powers, the scale-free topology β was selected as soft threshold power for **(A)** OA and **(B)** SCOS groups, respectively. OA, obstructive azoospermia; SCOS, Sertoli cell-only syndrome.

Supplementary Figure 6 Differential eigengene network analysis based on the OA and SCOS groups. The dendrograms of consensus module eigengenes within the **(A)** OA and **(B)** SCOS groups. The adjacencies of consensus module eigengenes within the **(C)** OA and **(D)** SCOS groups. Red and blue in the heatmap show high and low adjacencies, respectively. **(E)** The preservation network for the OA and SCOS groups. **(F)** Bar graph indicating the preservation degree of the consensus eigengenes. OA, obstructive azoospermia; SCOS, Sertoli cell-only syndrome.

Supplementary Figure 7 Functional enrichment of genes within the SCOS-specific green, red, or cyan module. SCOS, Sertoli cell-only syndrome.

Supplementary Figure 8 Functional enrichment of genes within SCOS-specific brown, lightcyan, greenyellow, salmon, or gray60 module. SCOS, Sertoli cell-only syndrome.

Supplementary Figure 9 The forest plots showing the significant associations between hub genes and immune cells without CD56^bright^ natural killer cell.
