# Supplementary figures and images for: Aberrant Gene Expression Profiling in Men With Sertoli Cell-Only Syndrome
Source: Front Immunol. 2022 Jun 27;13:821010. doi: 10.3389/fimmu.2022.821010 (PMC9273009; doi:10.3389/fimmu.2022.821010)

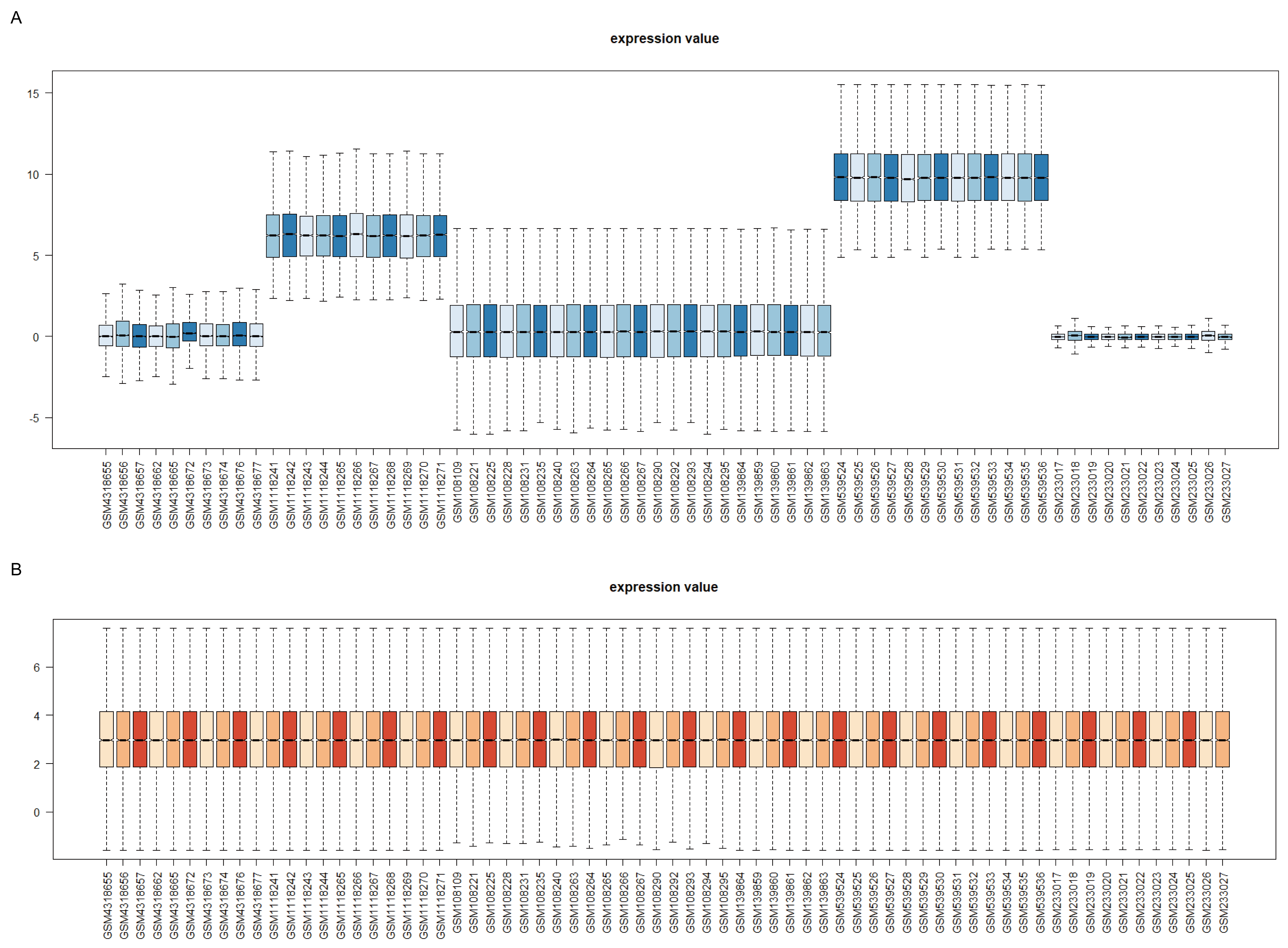

Supplement: Supplementary Figure 1 — Bar graphs depicting expression values of SCOS and OA testicular tissue samples (A) prior to and (B) after normalization. OA, obstructive azoospermia; SCOS, Sertoli cell-only syndrome. [file Image_1.tif]

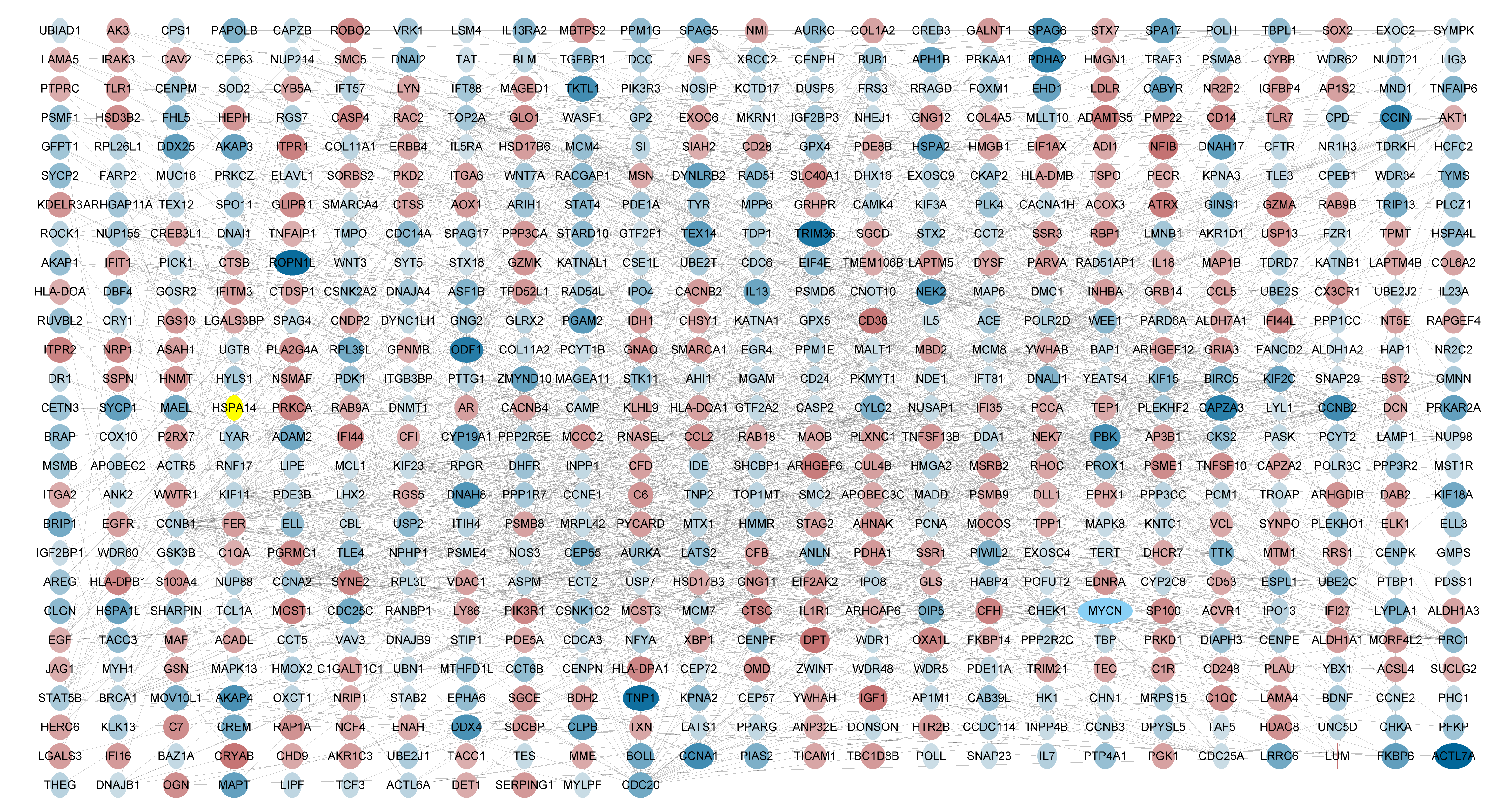

Supplement: Supplementary Figure 2 — PPI network of DEGs. Red and blue dots represent gene expression levels corresponding to upregulated and downregulated expression. Node size varies scaled proportional to the connectivity of the node and the edge indicates direct correlation. DEGs: differentially expressed genes; PPI: protein-protein interaction. [file Image_2.tif]

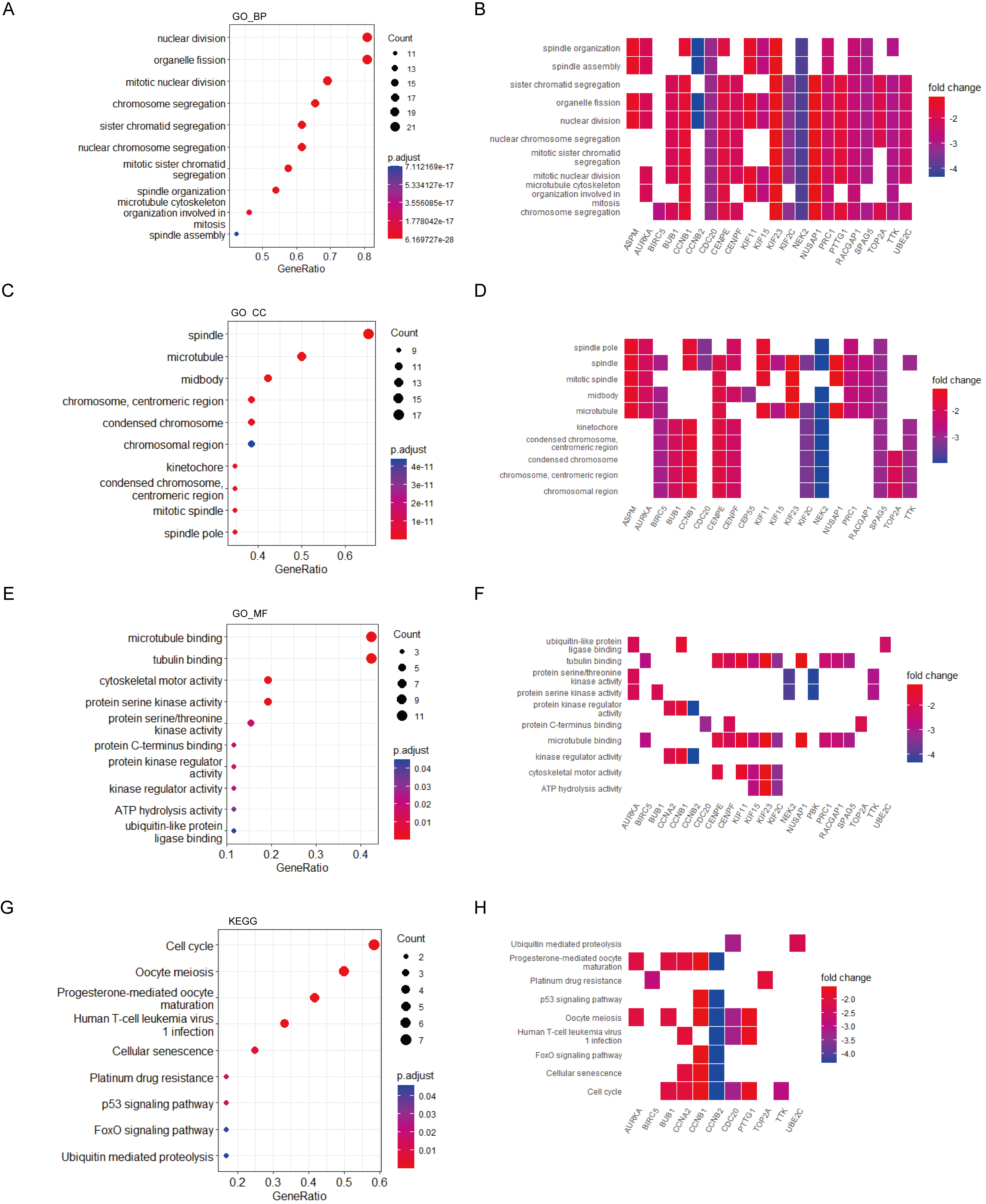

Supplement: Supplementary Figure 3 — Functional enrichment of genes within module 1. GO-BP analysis for genes in module 1 showing the significant terms through (A) a bubble plot and specific genes associated with these terms through (B) a heatmap. GO-CC analysis for the genes in module 1 showing the significant terms through (C) a bubble plot and specific genes associated with these terms through (D) a heatmap. GO-MF analysis for the genes in module 1 showing the significant terms through (E) a bubble plot and specific genes associated with these terms through (F) a heatmap. KEGG function pathway analysis of genes in module 1 indicating the enriched pathways through (G) a bubble plot and specific genes associated with these function pathways through (H) a heatmap. BP: biological process; CC: cellular component; DEGs: differentially expressed genes; GO: Gene Ontology; KEGG: Kyoto Encyclopedia of Genes and Genomes; MF: molecular function. [file Image_3.tif]

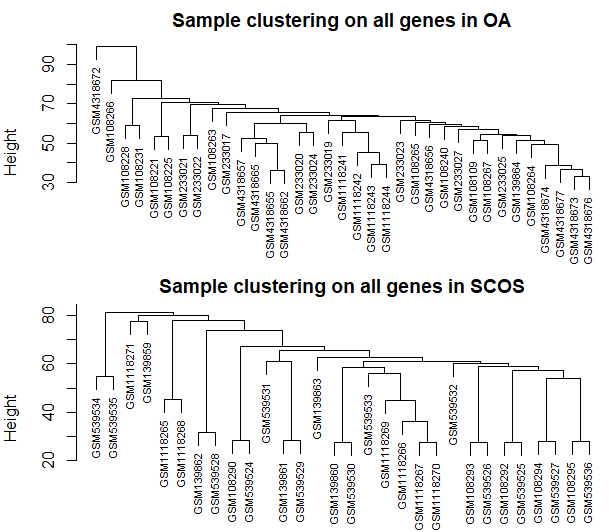

Supplement: Supplementary Figure 4 — Hierarchical cluster analysis of samples in the OA and SCOS testicular tissue samples. OA, obstructive azoospermia; SCOS, Sertoli cell-only syndrome. [file Image_4.tiff]

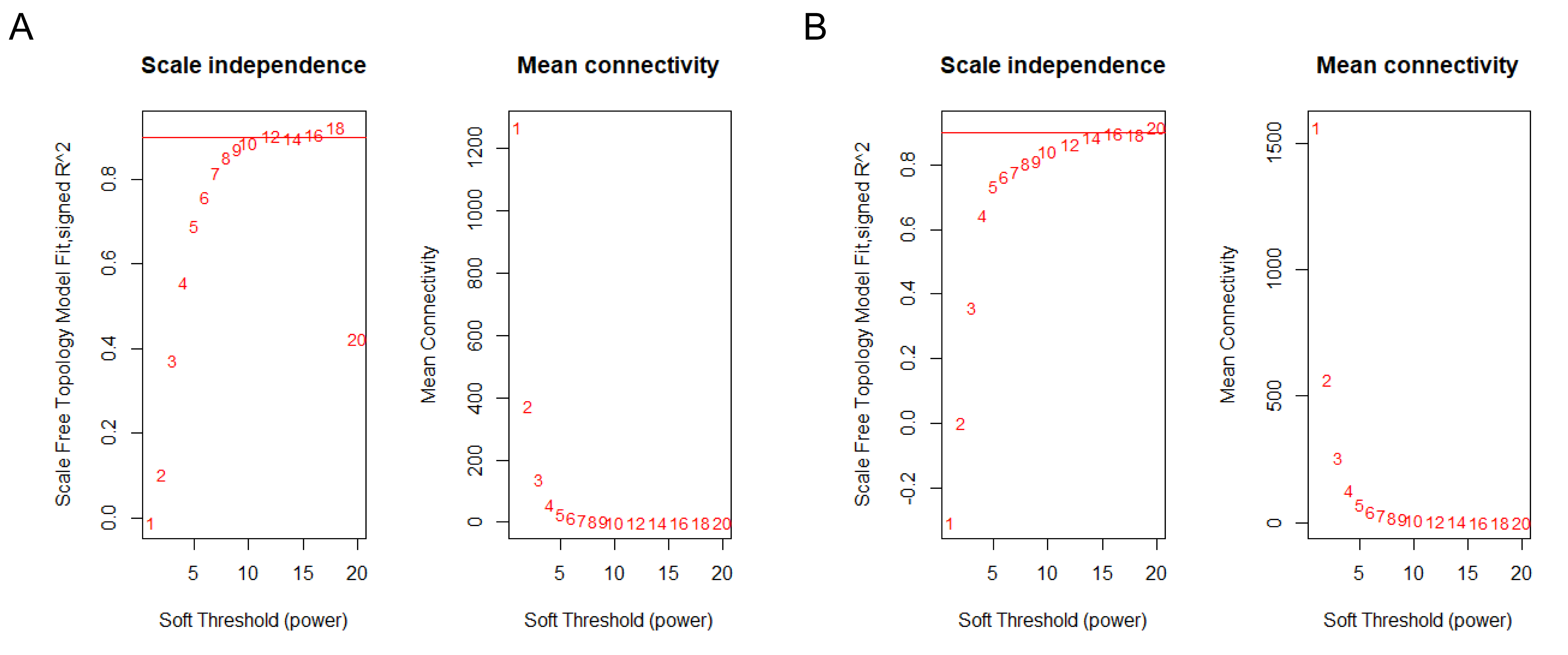

Supplement: Supplementary Figure 5 — After network topology analysis for soft-threshold powers, the scale-free topology β was selected as soft threshold power for (A) OA and (B) SCOS groups, respectively. OA, obstructive azoospermia; SCOS, Sertoli cell-only syndrome. [file Image_5.tif]

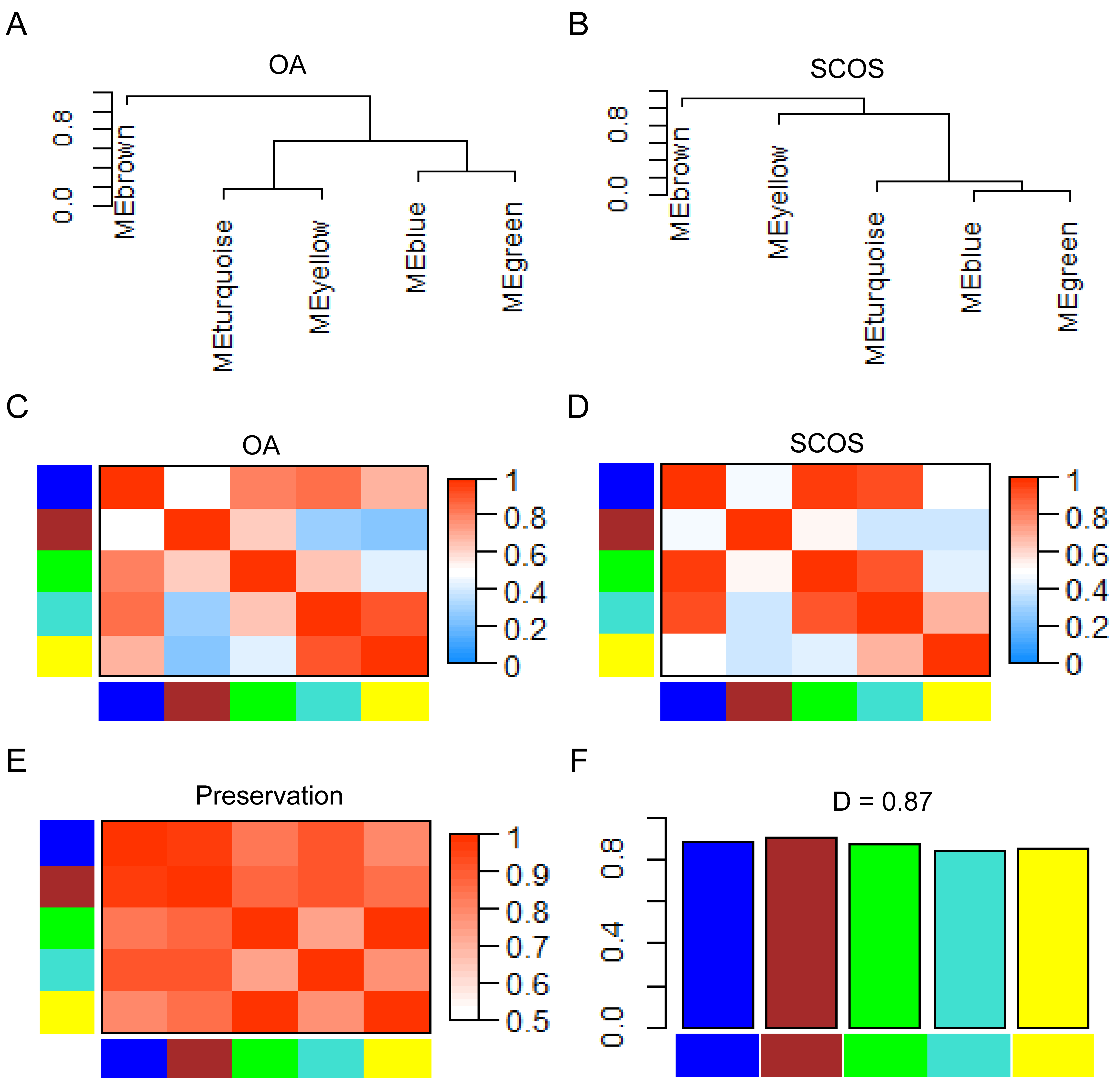

Supplement: Supplementary Figure 6 — Differential eigengene network analysis based on the OA and SCOS groups. The dendrograms of consensus module eigengenes within the (A) OA and (B) SCOS groups. The adjacencies of consensus module eigengenes within the (C) OA and (D) SCOS groups. Red and blue in the heatmap show high and low adjacencies, respectively. (E) The preservation network for the OA and SCOS groups. (F) Bar graph indicating the preservation degree of the consensus eigengenes. OA, obstructive azoospermia; SCOS, Sertoli cell-only syndrome. [file Image_6.tif]

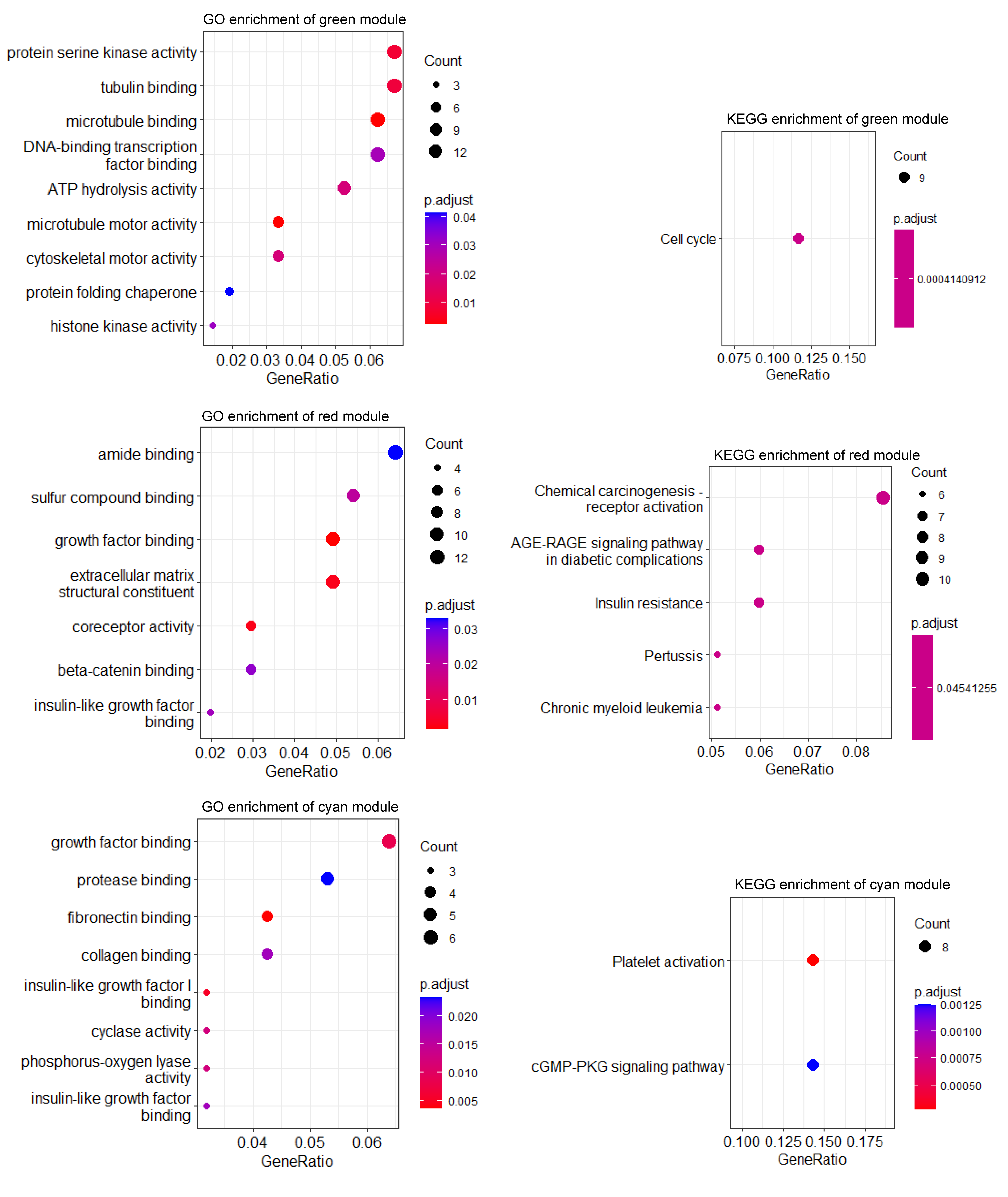

Supplement: Supplementary Figure 7 — Functional enrichment of genes within the SCOS-specific green, red, or cyan module. SCOS, Sertoli cell-only syndrome. [file Image_7.tif]

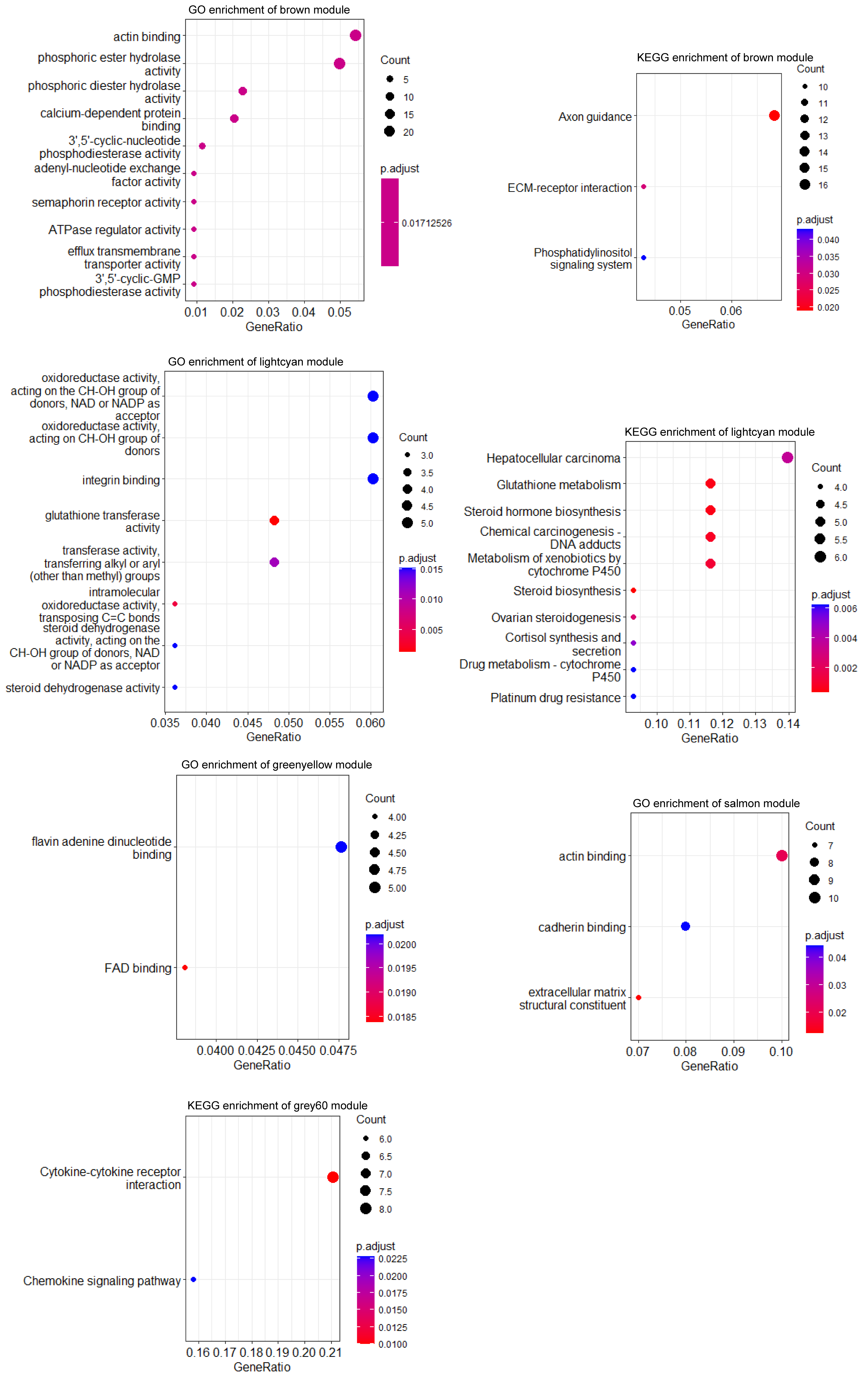

Supplement: Supplementary Figure 8 — Functional enrichment of genes within SCOS-specific brown, lightcyan, greenyellow, salmon, or gray60 module. SCOS, Sertoli cell-only syndrome. [file Image_8.tif]

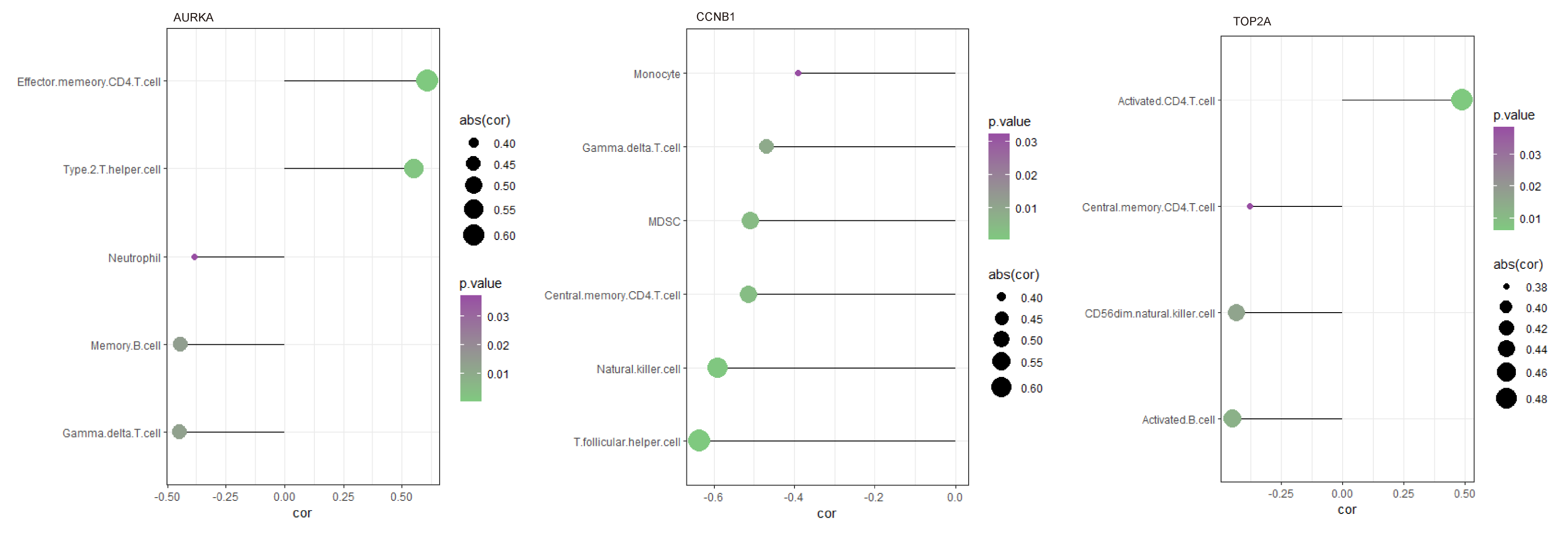

Supplement: Supplementary Figure 9 — The forest plots showing the significant associations between hub genes and immune cells without CD56bright natural killer cell. [file Image_9.tif]
