# Supplementary material for: Aberrant Gene Expression Profiling in Men With Sertoli Cell-Only Syndrome
Source: Front Immunol. 2022 Jun 27;13:821010. doi: 10.3389/fimmu.2022.821010 (PMC9273009; doi:10.3389/fimmu.2022.821010)
Supplement: Supplementary file 10 [file Table_1.docx]

**Table S1. The detailed information of datasets used in this study.**

| **GEO ID** | **Platform** | **OA** | **SCOS** | **Year** | **Country** |
| --- | --- | --- | --- | --- | --- |
| **GSE45885** | GPL6244 | 4 | 7 | 2013 | Norway |
| **GSE4797** | GPL2891 | 12 | 5 | 2006 | Germany |
| **GSE6023** | GPL2891 | 1 | 5 | 2006 | Germany |
| **GSE21613** | GPL2891 | 0 | 13 | 2010 | Germany |
| **GSE9210** | GPL887 | 11 | 0 | 2007 | Japan |
| **GSE145467** | GPL4133 | 10 | 0 | 2020 | Slovenia |

Abbreviations: OA, obstructive azoospermia; SCOS, Sertoli cell–only syndrome.
