# Supplementary material for: Aberrant Gene Expression Profiling in Men With Sertoli Cell-Only Syndrome
Source: Front Immunol. 2022 Jun 27;13:821010. doi: 10.3389/fimmu.2022.821010 (PMC9273009; doi:10.3389/fimmu.2022.821010)
Supplement: Supplementary file 11 [file Table_2.docx]

**Table S2. Hub genes identified using the Cytohubba plugin of Cytoscape.**

| **Rank** | **Ranking methods in the CytoHubba plugin** | | | | | | | | | | |
| --- | --- | --- | --- | --- | --- | --- | --- | --- | --- | --- | --- |
|  | Betweenness | BottleNeck | Closeness | Degree | DMNC | EcCentricity | EPC | MCC | MNC | Radiality | Sress |
| 1 | AKT1 | STAT5B | CCNB1 | CCNB1 | NEK2 | TBP | AURKA | TOP2A | NEK2 | AKT1 | CCNB1 |
| 2 | CCNB1 | CCNB1 | AKT1 | CCNA2 | CDCA3 | ACTL6A | KIF23 | ASPM | CDCA3 | CCNB1 | AKT1 |
| 3 | AR | PIK3R1 | AURKA | TOP2A | KIF15 | SMARCA4 | KIF11 | CCNA2 | KIF15 | BRCA1 | STIP1 |
| 4 | PIK3R1 | AKT1 | CCNA2 | CCNB2 | KIF2C | RUVBL2 | PBK | CDC20 | KIF2C | AURKA | AURKA |
| 5 | AURKA | TOP2A | TOP2A | CDC20 | CENPF | AKT1 | PRC1 | CCNB2 | CENPF | AR | BRCA1 |
| 6 | STAT5B | AR | CCNB2 | BUB1 | CEP55 | TEP1 | CENPF | KIF11 | CEP55 | YWHAH | HSPA1L |
| 7 | BRCA1 | TBP | CDC20 | KIF11 | SPAG5 | TERT | TOP2A | BUB1 | SPAG5 | STAT5B | TOP2A |
| 8 | EGFR | EGFR | BRCA1 | AURKA | PTTG1 | GSK3B | ASPM | CCNB1 | PTTG1 | PCNA | NUP214 |
| 9 | TERT | BRCA1 | BUB1 | ASPM | RACGAP1 | GTF2F1 | CEP55 | KIF2C | RACGAP1 | GSK3B | NUP98 |
| 10 | GSK3B | PCNA | KIF11 | UBE2C | PBK | CCNA2 | BUB1 | NUSAP1 | PBK | TOP2A | HSPA2 |
| 11 | PCNA | TERT | PCNA | NUSAP1 | BIRC5 | NFYA | CENPE | AURKA | BIRC5 | CCNA2 | NUP155 |
| 12 | TBP | RUVBL2 | UBE2C | TTK | CKAP2 | DR1 | CCNA2 | TTK | CKAP2 | TERT | NUP88 |
| 13 | RUVBL2 | GSK3B | AR | BIRC5 | ECT2 | PRKCA | TTK | BIRC5 | ECT2 | YWHAB | PIK3R1 |
| 14 | LYN | YWHAB | ASPM | AKT1 | PRC1 | GTF2A2 | MCM4 | CEP55 | PRC1 | EGFR | HSPA4L |
| 15 | WDR5 | LYN | PIK3R1 | CENPE | TTK | TBPL1 | SPAG5 | CENPF | TTK | PIK3R1 | STAT5B |
| 16 | XBP1 | PRKCA | YWHAH | KIF23 | NUSAP1 | POLR2D | CDC20 | NEK2 | NUSAP1 | CCNB2 | AR |
| 17 | MAPK8 | NUP98 | STAT5B | CEP55 | KIF23 | TAF5 | CCNB2 | UBE2C | KIF23 | CCNE1 | CCNB2 |
| 18 | CCNA2 | HK1 | BIRC5 | PRC1 | CENPE | ACTR5 | MCM7 | PBK | CENPE | PRKCA | CCT5 |
| 19 | HNMT | CFTR | NUSAP1 | CENPF | FOXM1 | YEATS4 | CCNB1 | PRC1 | FOXM1 | CDC20 | GSK3B |
| 20 | NFYA | MAPK8 | EGFR | KIF2C | TACC3 | CUL4B | ESPL1 | SPAG5 | TACC3 | ITGA6 | DNAJB1 |
| 21 | PRKCA | EIF4E | CHEK1 | PBK | ESPL1 | MORF4L2 | BIRC5 | CENPE | ESPL1 | UBE2C | TERT |
| 22 | MAOB | WDR5 | TTK | CDC6 | ANLN | ANP32E | KIF15 | KIF23 | ANLN | CHEK1 | CCNA2 |
| 23 | TOP2A | DR1 | CENPE | PIK3R1 | KIF18A | PDE3B | FOXM1 | PTTG1 | KIF18A | CCNA1 | LYN |
| 24 | AOX1 | GFPT1 | GSK3B | SPAG5 | ASPM | AURKA | UBE2C | CDCA3 | ASPM | KIF11 | VDAC1 |
| 25 | PRKAR2A | LMNB1 | PRC1 | NEK2 | SMC2 | ARHGDIB | NEK2 | KIF15 | SMC2 | BUB1 | EIF4E |
| 26 | NUP98 | NFYA | KIF23 | PCNA | AURKA | RBP1 | PTTG1 | RACGAP1 | AURKA | PRKCZ | TBP |
| 27 | VDAC1 | XBP1 | CEP55 | EGFR | ARHGAP11A | YWHAH | CDCA3 | ESPL1 | ARHGAP11A | PPP2R5E | YWHAH |
| 28 | YWHAB | LGALS3 | CCNE1 | PTTG1 | PLK4 | RAC2 | KIF2C | FOXM1 | PLK4 | BIRC5 | RUVBL2 |
| 29 | RHOC | HNMT | PBK | BRCA1 | CKS2 | MKRN1 | NUSAP1 | ECT2 | CKS2 | ASPM | EGFR |
| 30 | PTPRC | MAOB | CENPF | MCM4 | HMMR | GRB14 | PCNA | CKS2 | HMMR | NUSAP1 | XBP1 |
| 31 | LMNB1 | GNG2 | KIF2C | CDCA3 | PSMB8 | MST1R | CHEK1 | CKAP2 | PSMB8 | LYN | CCT2 |
| 32 | YWHAH | CHEK1 | YWHAB | RACGAP1 | PSMA8 | NOS3 | CDC6 | SMC2 | PSMA8 | PRC1 | GNAQ |
| 33 | HK1 | IL13 | CCNA1 | MCM7 | PSMD6 | ITPR1 | CCNA1 | MCM4 | PSMD6 | CDC25C | PCNA |
| 34 | PSMB8 | NMI | NEK2 | KIF15 | PSME1 | EDNRA | CDC25A | MCM7 | PSME1 | CENPE | WDR5 |
| 35 | ITGA6 | PRKCZ | TERT | CHEK1 | KIF11 | PLEKHF2 | CKAP2 | ANLN | KIF11 | KIF23 | YWHAB |
| 36 | HSD17B6 | AOX1 | SPAG5 | ESPL1 | UBE2C | PARVA | ECT2 | HMMR | UBE2C | CEP55 | NFYA |
| 37 | IL13 | GMNN | PRKCA | CDC25A | PSMB9 | PPP2R5E | AKT1 | TACC3 | PSMB9 | CKS2 | PRKCA |
| 38 | PRKCZ | PRKAR2A | PTTG1 | FOXM1 | PSME4 | MCL1 | RACGAP1 | CDC6 | PSME4 | LMNB1 | RHOC |
| 39 | ITPR1 | AURKA | CDC6 | CCNA1 | BUB1 | RRAGD | BRCA1 | CHEK1 | BUB1 | TTK | BIRC5 |
| 40 | SMARCA4 | MCL1 | CKS2 | AR | IFIT1 | YWHAB | SMC2 | KIF18A | IFIT1 | NMI | ITPR1 |
| 41 | MCL1 | PSMB8 | CDCA3 | SMC2 | BST2 | AR | GMNN | GMNN | BST2 | PBK | PRKCZ |
| 42 | DCN | IFI35 | ITGA6 | CKS2 | RNASEL | PRKCZ | NUP155 | PSMB8 | RNASEL | ERBB4 | PTPRC |
| 43 | BIRC5 | PARD6A | FOXM1 | NUP214 | IFI27 | CYBB | CKS2 | FZR1 | IFI27 | FOXM1 | MAPK8 |
| 44 | GTF2F1 | STAT4 | MCM4 | PRKCA | IFITM3 | PFKP | PIK3R1 | PSMB9 | IFITM3 | NEK2 | HNMT |
| 45 | CCNB2 | SMARCA4 | CDC25C | ECT2 | IFI35 | NCF4 | NUP88 | PSME4 | IFI35 | CENPF | ECT2 |
| 46 | EIF4E | MAF | RACGAP1 | NUP155 | OIP5 | NRIP1 | NUP214 | PSMA8 | OIP5 | KIF2C | PSMB8 |
| 47 | CD36 | VDAC1 | KIF15 | NUP88 | PKMYT1 | TCL1A | NUP98 | PSMD6 | PKMYT1 | SPAG5 | MAOB |
| 48 | ECT2 | GTF2F1 | MCM7 | LYN | CDC20 | ITGA6 | EGFR | PSME1 | CDC20 | RACGAP1 | AOX1 |
| 49 | GOSR2 | NUP88 | LYN | GMNN | TROAP | BRCA1 | CCNE1 | ARHGAP11A | TROAP | PTTG1 | MCL1 |
| 50 | GNAQ | HSPA4L | GMNN | NUP98 | BRIP1 | PIK3R1 | HMMR | PLK4 | BRIP1 | TYMS | HK1 |
| 51 | TYMS | PTPRC | PRKCZ | PSMB8 | MCM8 | SOD2 | YWHAB | CCNA1 | MCM8 | CDCA3 | CDC6 |
| 52 | CFTR | HSD17B6 | TYMS | YWHAB | TOP2A | KIF23 | FZR1 | TYMS | TOP2A | MST1R | GMNN |
| 53 | NUP214 | DCN | ESPL1 | CKAP2 | TYMS | KIF11 | ANLN | NUP214 | TYMS | KIF15 | SMARCA4 |
| 54 | IGF1 | DNMT1 | LMNB1 | RAD51 | FZR1 | PBK | CCNB3 | NUP155 | FZR1 | FZR1 | IDE |
| 55 | IFIT1 | PFKP | ECT2 | GSK3B | CCNB2 | PRC1 | PRKCA | NUP88 | CCNB2 | EDNRA | LMNB1 |
| 56 | GMNN | ITGA6 | NUP214 | ANLN | CFTR | CENPF | PSME4 | CDC25A | CFTR | GMNN | DCN |
| 57 | STIP1 | BIRC5 | FZR1 | MAPK8 | ASF1B | TOP2A | CDC25C | CCNB3 | ASF1B | BLM | ITGA6 |
| 58 | IL18 | CCNA1 | CDC25A | CCNE1 | WEE1 | ASPM | PSMD6 | CDC25C | WEE1 | MCL1 | HSPA14 |
| 59 | ERBB4 | GNAQ | NUP155 | HMMR | MCM4 | CEP55 | TYMS | PCNA | MCM4 | ECT2 | PRKAR2A |
| 60 | GFPT1 | NUSAP1 | NUP88 | FZR1 | AKR1C3 | BUB1 | PSMB9 | NUP98 | AKR1C3 | MCM4 | HSD17B6 |
| 61 | CETN3 | OIP5 | NUP98 | TYMS | AKR1D1 | ECT2 | BLM | OIP5 | AKR1D1 | IL5 | GOSR2 |
| 61 | NUP155 | STIP1 | NMI | CDC25C | CYP19A1 | RACGAP1 | PSMB8 | CFTR | CYP19A1 | MCM7 | IFIT1 |
| 63 | NUP88 | CD36 | BLM | STAT5B | HSD3B2 | ERBB4 | EGF | IFIT1 | HSD3B2 | KPNA2 | TGFBR1 |
| 64 | DNMT1 | CETN3 | HMMR | YWHAH | NMI | EGFR | STAT5B | BST2 | NMI | RAC2 | IGF1 |
| 65 | GNG2 | TGFBR1 | PPP2R5E | EGF | MCM7 | EGF | YWHAH | IFI35 | MCM7 | CDC6 | ERBB4 |
| 66 | IDE | USP7 | ERBB4 | IFIT1 | HSD17B3 | ARHGAP11A | RAD51 | RNASEL | HSD17B3 | HMMR | CDC20 |
| 67 | IL5 | GOSR2 | MAPK8 | BLM | HSD17B6 | CENPE | TACC3 | IFI27 | HSD17B6 | IGF1 | GFPT1 |
| 68 | LGALS3 | RHOC | KPNA2 | EIF4E | ITGB3BP | TTK | CFTR | IFITM3 | ITGB3BP | RRAGD | CCNA1 |
| 69 | TGFBR1 | IDE | CCNB3 | PRKCZ | EIF4E | ASF1B | PSMA8 | CCNE1 | EIF4E | IL5RA | EDNRA |
| 70 | RPGR | YWHAH | SMC2 | ITGA6 | SHCBP1 | MCM4 | AR | WEE1 | SHCBP1 | TEP1 | CDC25A |
| 71 | CDC6 | ITGB3BP | EDNRA | CCNB3 | DBF4 | SPAG5 | PSME1 | PKMYT1 | DBF4 | MAPK8 | RRAGD |
| 72 | PFKP | CCNA2 | OIP5 | PSMB9 | CENPH | CDC20 | WEE1 | BRCA1 | CENPH | SMARCA4 | RGS5 |
| 73 | UBE2C | IFIT1 | IGF1 | PSME4 | CENPK | CCNB2 | LYN | RAD51 | CENPK | PPP1CC | SMC2 |
| 74 | HSPA1L | TYMS | WEE1 | CFTR | SYMPK | MCM7 | OIP5 | BLM | SYMPK | ESPL1 | UBE2C |
| 75 | ACTL6A | RPGR | MST1R | TACC3 | DDX4 | CCNB1 | KIF18A | CCNE2 | DDX4 | CDC25A | CHEK1 |
| 76 | ROCK1 | IL5 | RAC2 | IGF1 | MOV10L1 | ESPL1 | UBE2S | ASF1B | MOV10L1 | NUP214 | ACTL6A |
| 77 | POLR2D | PDE3B | IL5 | UBE2S | HSPA2 | PLK4 | ITGA6 | POLH | HSPA2 | NCF4 | IL13 |
| 78 | BDNF | ITPR1 | MCL1 | OIP5 | HSPA14 | BIRC5 | ARHGAP11A | TROAP | HSPA14 | NUP98 | GTF2F1 |
| 79 | PIAS2 | CAPZB | UBE2S | LMNB1 | HSPA4L | KIF15 | CCNE2 | BRIP1 | HSPA4L | NUP155 | KIF11 |
| 80 | NMI | UBE2C | RAD51 | CBL | IPO8 | TACC3 | PLK4 | MCM8 | IPO8 | NUP88 | GNG2 |
| 81 | CHEK1 | KPNA2 | EGF | PSMA8 | NEK7 | FOXM1 | GSK3B | EGFR | NEK7 | CCNB3 | TYMS |
| 82 | USP7 | UBE2S | PKMYT1 | PSMD6 | SP100 | UBE2C | PKMYT1 | PIK3R1 | SP100 | GTF2F1 | IL5 |
| 83 | CCNA1 | CBL | CKAP2 | PSME1 | CCNB3 | NEK2 | MAPK8 | EGF | CCNB3 | OIP5 | RAP1A |
| 84 | HMGB1 | PTP4A1 | PRKAR2A | RHOC | CCNA2 | PTTG1 | PRKCZ | STAT5B | CCNA2 | RAD51 | BUB1 |
| 85 | DYNC1LI1 | HMGB1 | TACC3 | HSD17B6 | CCNE2 | CKS2 | EIF4E | AKT1 | CCNE2 | WEE1 | IL18 |
| 86 | PPP1CC | CENPN | IL5RA | RAC2 | CDC25A | CDCA3 | ASF1B | KPNA2 | CDC25A | EGF | HLA-DPB1 |
| 87 | SMC2 | HLA-DPA1 | SMARCA4 | GNAQ | KPNA2 | CKAP2 | KPNA2 | LYN | KPNA2 | ARHGDIB | HLA-DPA1 |
| 88 | AK3 | ROCK1 | NCF4 | KIF18A | FANCD2 | KIF2C | ERBB4 | NMI | FANCD2 | PPP3CC | POLR2D |
| 89 | CDC20 | MAPT | PPP1CC | KPNA2 | FKBP6 | NUSAP1 | CBL | AKR1C3 | FKBP6 | PFKP | ACOX3 |
| 90 | HSPA2 | POLR2D | EIF4E | VDAC1 | PIWIL2 | FZR1 | NMI | AKR1D1 | PIWIL2 | CSNK1G2 | ASPM |
| 91 | OIP5 | AK3 | TEP1 | IL18 | MAEL | TYMS | PSMF1 | CYP19A1 | MAEL | PKMYT1 | CFTR |
| 92 | KPNA2 | AKAP1 | CCNE2 | IL13 | POLH | PCNA | HSPA2 | HSD3B2 | POLH | ITPR1 | OIP5 |
| 93 | DR1 | AKAP3 | RRAGD | WEE1 | CCNB1 | CHEK1 | POLH | UBE2S | CCNB1 | UBE2S | KPNA2 |
| 93 | NT5E | BUB1 | CBL | STIP1 | GMNN | CCNE1 | HSPA1L | PSMF1 | GMNN | POLR2D | INPP4B |
| 95 | CACNB2 | ERBB4 | ANLN | DCN | PSMF1 | CDC6 | MCM8 | PRKCA | PSMF1 | EIF4E | CD36 |
| 95 | TRAF3 | DYNC1LI1 | RAD51AP1 | DNAJB1 | GTF2F1 | OIP5 | TERT | EIF4E | GTF2F1 | SOD2 | MCM4 |
| 95 | AKAP1 | PIAS2 | POLH | HSPA1L | COL11A1 | CDC25C | IGF1 | CBL | COL11A1 | GRB14 | PFKP |
| 98 | AKAP3 | MCM7 | STAG2 | XBP1 | COL11A2 | LMNB1 | HSPA4L | YWHAH | COL11A2 | MAPT | LGALS3 |
| 99 | PMP22 | SMC2 | GNG2 | TBP | CCNE1 | CCNA1 | PPP2R5E | YWHAB | CCNE1 | CYBB | NMI |
| 100 | CD28 | RAD51 | ITPR1 | ACTL6A | CDC25C | NMI | UBE2T | FANCD2 | CDC25C | SMC2 | SYMPK |
