# Supplementary material for: Aberrant Gene Expression Profiling in Men With Sertoli Cell-Only Syndrome
Source: Front Immunol. 2022 Jun 27;13:821010. doi: 10.3389/fimmu.2022.821010 (PMC9273009; doi:10.3389/fimmu.2022.821010)
Supplement: Supplementary file 12 [file Table_3.docx]

**Table S3. Upstream transcription factors predicted based on the downregulated genes.**

| **Rank** | **Transcription Factor** | **Hypergeometric *P* value** | **Enriched target genes** |
| --- | --- | --- | --- |
| 1 | E2F4 | 1.04E-22 | 108 |
| 2 | FOXM1 | 2.78E-08 | 22 |
| 3 | NFYA | 1.14E-07 | 168 |
| 4 | NFYB | 0.000001355 | 245 |
| 5 | E2F6 | 0.000006368 | 215 |
| 6 | SIN3A | 0.0001263 | 86 |
| 7 | CREB1 | 0.001701 | 98 |
| 8 | NRF1 | 0.002978 | 121 |
| 9 | BRCA1 | 0.00575 | 191 |
| 10 | KLF4 | 0.0177 | 65 |
