# Supplementary material for: Aberrant Gene Expression Profiling in Men With Sertoli Cell-Only Syndrome
Source: Front Immunol. 2022 Jun 27;13:821010. doi: 10.3389/fimmu.2022.821010 (PMC9273009; doi:10.3389/fimmu.2022.821010)
Supplement: Supplementary file 13 [file Table_4.docx]

**Table S4. Upstream protein kinase predicted based on the downregulated genes.**

| **Rank** | **Protein kinase** | **Hypergeometric *P* value** | **Enriched target genes** |
| --- | --- | --- | --- |
| 1 | CSNK2A1 | 3.58E-22 | 43 |
| 2 | CDK1 | 1.26E-21 | 44 |
| 3 | MAPK14 | 2.95E-18 | 33 |
| 4 | CDK4 | 2.21E-17 | 18 |
| 5 | CDC2 | 1.09E-16 | 22 |
| 6 | CK2ALPHA | 8.87E-16 | 20 |
| 7 | ATM | 2.00E-14 | 19 |
| 8 | CDK2 | 2.79E-14 | 43 |
| 9 | GSK3B | 1.05E-11 | 37 |
| 10 | MAPK1 | 1.15E-11 | 23 |
